# Supplementary material for: Network Proximity-Based Drug Repurposing Strategy for Early and Late Stages of Primary Biliary Cholangitis
Source: Biomedicines. 2022 Jul 13;10(7):1694. doi: 10.3390/biomedicines10071694 (PMC9312896; doi:10.3390/biomedicines10071694)
Supplement: Supplementary file 1 [file biomedicines-10-01694-s001.zip › biomedicines-1718019-supplementary.pdf]

List of Supplementary Material

Contents

Table S1: Input Seed Genes. PMID = PubMed unique identifier. 1

Table S2: Over-representation analysis (ORA) from Seed Genes results for drugs. Results are stratified by disease stages (Label column). 32

Table S3: Over-representation analysis (ORA) from Likely Positives (LP) results for drugs. Results are stratified by disease stages (Label column). 39

Table S4: Over-representation analysis (ORA) from Seed genes results for pathways. Results are stratified by disease stages (Label column). 42

Table S5: Over-representation analysis (ORA) from Likely Positives (LP) results for pathways. Results are stratified by disease stages (Label column). 45

Supplementary File S1: Detailed Methodology. 52

Supplementary File S2: l-threonine Webgestalt results. 52

Supplementary File S3: l-serine Webgestalt results. 52

Supplementary File S4: l-lysine Webgestalt results. 52

Supplementary File S5: PA45261 Webgestalt results. 52

Supplementary File S6: PA164712966 Webgestalt results. 52

Supplementary File S7: PA164712732 Webgestalt results. 52

Table S1: Input Seed Genes. **PMID** = PubMed unique identifier.

GENE	Stage	Approved	Suggested symbol	Score final	PMID
A2BP1	US	False	RBFOX1	0.64	20153395
ABCA1	US	True	ABCA1	0.01	28660384
ABCA5	Early Stages	True	ABCA5	0.33	19669248
ABCB1	Late Stages	True	ABCB1	0.32	12763363;15690482;16584389; 27913155
ABCB11	US	True	ABCB11	0.64	12763363;20857261;14999697;25392597
ABCB4	US	True	ABCB4	0.64	23704821;30682444;9126799;12763363;17295178;18671305;18662272;26324191;2

					4620780;20040336;14999697
ABCC1	US	True	ABCC1	0.01	12763363
ABCC2	US	True	ABCC2	0.52	15770136;12763363;14568249;15542527
ABCC3	US	True	ABCC3	0.02	14568249;12763363
ABCC4	US	True	ABCC4	0.01	17696930
ABCC6	US	True	ABCC6	0.01	14568249
ABCD1	US	True	ABCD1	0.64	24734033
ABCG2	US	True	ABCG2	0.3	15542527
ABO	US	True	ABO	0.01	4211827
ACE	US	True	ACE	0.21	19389807;19119484
ACE2	US	True	ACE2	0.2	17532087
ACTA1	Late Stages	True	ACTA1	0.32	28889961
ACTB	Late Stages	True	ACTB	0.32	12791319
ADA	Late Stages	True	ADA	0.32	27913155
ADIRF	Early Stages	True	ADIRF	0.33	18422935
ADRB2	US	True	ADRB2	0.64	31948396
AEN	Early Stages	True	AEN	0.33	27913155
AESTPC1	Early Stages	False	#N/A	0.33	18422935
AGBL2	US	True	AGBL2	0.01	30807792
AGO2	US	True	AGO2	0.01	23224974
AGT	US	True	AGT	0.2	17532087
AGTRL1	Early Stages	False	APLNR	0.33	18422935
AGXT2	Early Stages	True	AGXT2	0.33	18422935
AHSG	US	True	AHSG	0.64	23958878
AIRE	US	True	AIRE	0.01	11343230
AKAP11	US	True	AKAP11	0.64	26842849
ALB	US	True	ALB	0.35	31419573;31430975;31807995;30026087;31651244;29380062
ALPP	US	True	ALPP	0.03	29193593;14687829;31714880
ALS2CR19	Early Stages	False	PARD3B	0.33	18422935
AMBP	Early Stages	True	AMBP	0.33	27913155
AMICA1	Late Stages	False	JAML	0.32	27913155
ANGPTL4	US	True	ANGPTL4	0.64	29297981
ANKRD36B	Late Stages	True	ANKRD36B	0.32	11488641
ANXA11	US	True	ANXA11	0.01	23152861
ANXA2	US	True	ANXA2	0.01	28476852

AP2M1	Early Stages	True	AP2M1	0.33	18422935
APEX1	US	True	APEX1	0.01	18671305
APOB	Early Stages	True	APOB	0.33	19669248
APOE	US	True	APOE	0.02	92520871;1592592
AQP4	US	True	AQP4	0.2	20451280
AREB6	Early Stages	False	ZEB1	0.33	16128947
AREG	US	True	AREG	0.01	30411380
ARF7	US	False	ARL14	0.64	19458352
ARG1	Early Stages	True	ARG1	0.33	27913155
ARG2	Late Stages	True	ARG2	0.32	11488641
ARHGAP31	US	True	ARHGAP31	0.1	21399635
ARHGAP4	Early Stages	True	ARHGAP4	0.33	18422935
ARID3A	US	True	ARID3A	0.64	28425483
ARL7	US	False	ARL4C	0.64	23958878
ARRB1	US	True	ARRB1	0.64	26267705
ASRGL1	US	True	ASRGL1	0.03	29193593;31714880;14687829
ATAT1	Late Stages	True	ATAT1	0.32	16128947
ATF1	Early Stages	True	ATF1	0.33	27913155
ATHS	US	False	#N/A	0.03	31714880;14687829;29193593
ATM	US	True	ATM	0.01	18456456
ATN1	US	True	ATN1	0.03	29158418;21169553;16636131
ATP12A	US	True	ATP12A	0.64	24734033
ATP1B1	Late Stages	True	ATP1B1	0.32	11488641
ATP5A1	US	False	ATP5F1A	0.64	24734033
ATRNL1	US	True	ATRNL1	0.03	14687829;29193593;31714880
ATXN2	US	True	ATXN2	0.64	22961000
AXUD1	US	False	CSRNP1	0.64	23958878
B2M	Early Stages	True	B2M	0.33	18422935
B3GAT1	US	True	B3GAT1	0.02	21735469;12654131
BANK1	US	True	BANK1	0.64	26000122
BAX	Late Stages	True	BAX	0.32	27913155
BC10	Late Stages	False	BLCAP	0.32	11488641
BCAP31	US	True	BCAP31	0.64	24734033
BCGF1	Late Stages	False	IL4	0.32	24734033
BCL2	Late Stages	True	BCL2	0.32	27913155;30253330;9855077;26429926;9141420
BCL-2	US	False	BCL2	0.64	30253330
BCL3	US	True	BCL3	0.01	24293610
BCR	US	True	BCR	0.01	18176868
BDNF	Early Stages	True	BDNF	0.33	16128947
BG1	Early Stages	False	ACSBG1	0.33	18422935

BGN	US	True	BGN	0.64	24734033
BHMT	US	True	BHMT	0.01	24620780
BIN2	Early Stages	True	BIN2	0.33	18422935
BRAP	US	True	BRAP	0.64	30992889
BRCA1	US	True	BRCA1	0.01	24947112
BRCC3	US	True	BRCC3	0.64	24734033
BST2	Late Stages	True	BST2	0.32	27913155
BTG3	US	True	BTG3	0.02	30807792;14687829
BTK	Early Stages	True	BTK	0.33	16128947
BTNL2	US	True	BTNL2	0.64	19458352
C1ORF94	US	False	C1orf94	0.64	26084578
C1QBP	Early Stages	True	C1QBP	0.33	27913155
C1QR1	US	False	CD93	0.64	23958878
C1R	Early Stages	True	C1R	0.33	27913155
C1S	Early Stages	True	C1S	0.33	27913155
C4A	US	True	C4A	0.02	7927254;3496684
C4AQO	US	False	#N/A	0.64	1936808
C4B_2	US	False	#N/A	0.64	7927254;3496684
C4BPA	Early Stages	True	C4BPA	0.33	27913155
C5	Early Stages	True	C5	0.33	27913155
C6	Early Stages	True	C6	0.33	27913155
C8A	Early Stages	True	C8A	0.33	27913155
C9	Early Stages	True	C9	0.33	27913155
C9ORF10	Early Stages	False	FAM120A	0.33	18422935
CA5AP1	Late Stages	True	CA5AP1	0.32	11488641
CACYBP	Late Stages	True	CACYBP	0.32	11488641
CADM1	Early Stages	True	CADM1	0.33	16128947
CALCOCO2	US	True	CALCOCO2	0.01	9230084
CALM1	Late Stages	True	CALM1	0.32	11488641
CAP2	Early Stages	True	CAP2	0.33	18422935
CAPSL	US	True	CAPSL	0.64	22936693;34033851
CARD11	Late Stages	True	CARD11	0.32	27913155
CAS	Late Stages	False	BCAR1 /// CSE1L	0.32	16128947
CASP8	Late Stages	True	CASP8	0.32	27913155
CAST	Late Stages	True	CAST	0.32	11488641
CAV2	Late Stages	True	CAV2	0.32	11488641
CCDC113	US	True	CCDC113	0.64	30259846
CCDC6	US	True	CCDC6	0.64	26084578
CCDC68	US	True	CCDC68	0.64	19458352
CCDC88B	US	True	CCDC88B	0.64	26394269
CCL11	US	True	CCL11	0.64	32363322;30583236
CCL13	Late Stages	True	CCL13	0.32	27913155
CCL14	Early Stages	True	CCL14	0.33	27913155
CCL18	US	True	CCL18	0.64	32363322
CCL19	US	True	CCL19	0.64	32363322
CCL2	US	True	CCL2	0.64	32363322
CCL20	US	True	CCL20	0.64	21092071
CCL23	Early Stages	True	CCL23	0.33	27913155

CCL24	US	True	CCL24	0.01	30583236
CCL25	US	True	CCL25	0.64	29297981
CCL26	US	True	CCL26	0.01	30583236
CCL27	US	True	CCL27	0.03	29193593;31714880;14687829
CCL3	Late Stages	True	CCL3	0.32	27913155
CCL3L1	Late Stages	True	CCL3L1	0.32	27913155
CCL4	Late Stages	True	CCL4	0.32	27913155
CCL5	US	True	CCL5	0.64	23958878;12126966
CCN2	US	True	CCN2	0.2	19371232
CCN6	US	True	CCN6	0.64	29297981
CCND2	Late Stages	True	CCND2	0.32	16128947
CCND3	Late Stages	True	CCND3	0.32	27913155
CCR2	US	True	CCR2	0.64	32363322
CCR5	US	True	CCR5	0.64	32363322;15566517;16721228
CCR6	US	True	CCR6	0.64	26084578;34033851
CCR7	US	True	CCR7	0.01	14517790
CCT3	Early Stages	True	CCT3	0.33	18422935
CCT5	Early Stages	True	CCT5	0.33	18422935
CD14	US	True	CD14	0.64	23958878;27860118;26970220;20653431
CD180	US	True	CD180	0.01	17448566
CD19	US	True	CD19	0.64	26429926;27784538;21092071
CD1C	Early Stages	True	CD1C	0.33	21092071
CD1D	US	True	CD1D	0.01	12360465
CD20	Early Stages	False	MS4A1	0.33	16128947
CD207	US	True	CD207	0.01	21092071
CD226	US	True	CD226	0.64	34033851
CD24	Late Stages	True	CD24	0.32	27913155
CD244	US	True	CD244	0.64	26084578
CD248	Early Stages	True	CD248	0.33	18422935
CD274	US	True	CD274	0.01	31359214
CD28	US	True	CD28	0.64	26347073
CD34	Late Stages	True	CD34	0.32	27913155
CD3D	Early Stages	True	CD3D	0.33	18422935
CD40	US	True	CD40	0.03	26388238;16448457;11689460
CD40L	US	False	CD40LG	0.64	30343705
CD40LG	US	True	CD40LG	0.04	21898485;9819192;16448457;29400703
CD47	Late Stages	True	CD47	0.32	27913155
CD48	US	True	CD48	0.64	32363322
CD49A	US	False	ITGA1	0.64	19333938
CD58	US	True	CD58	0.64	28425483
CD59	Late Stages	True	CD59	0.32	27913155

CD68	Late Stages	True	CD68	0.32	27913155
CD69	US	True	CD69	0.64	31948396
CD72	US	True	CD72	0.64	32363322
CD74	US	True	CD74	0.01	23913513
CD79A	US	True	CD79A	0.02	12360422;9802945
CD80	US	True	CD80	0.64	28425483;21399635;23000144;30643196;31027870
CD83	Late Stages	True	CD83	0.32	27913155
CD86	US	True	CD86	0.02	31027870;29312539
CD96	US	True	CD96	0.64	32363322
CD99	US	True	CD99	0.01	19638108
CDC2	US	False	CDK1 /// POLD1	0.64	23958878
CDC25C	Early Stages	True	CDC25C	0.33	18422935
CDC27	Early Stages	True	CDC27	0.33	16128947
CDH11	Early Stages	True	CDH11	0.33	16128947
CDH13	Early Stages	True	CDH13	0.33	16128947
CDH3	Early Stages	True	CDH3	0.33	16128947
CDH5	Late Stages	True	CDH5	0.32	27913155;18422935
CDH6	Early Stages	True	CDH6	0.33	16128947
CDK1	US	True	CDK1	0.64	32363322
CDK12	US	True	CDK12	0.1	21399635
CDK16	US	True	CDK16	0.64	24734033
CDK19	US	True	CDK19	0.64	29297981
CDK2AP1	US	True	CDK2AP1	0.01	29229992
CDK8	US	True	CDK8	0.64	29297981
CDKN1A	Late Stages	True	CDKN1A	0.32	27913155;29540861;11394648;18456456;22098537;18536059
CDKN1B	US	True	CDKN1B	0.02	24741631;30253330
CDKN2A	US	True	CDKN2A	0.04	31812332;11171828;18536059;18456456
CENPF	US	True	CENPF	0.64	32363322
CETN3	US	True	CETN3	0.64	23958878
CFB	Late Stages	True	CFB	0.32	11488641
CFHR4	Late Stages	True	CFHR4	0.32	11488641
CFP	US	True	CFP	0.64	24734033
CFTR	US	True	CFTR	0.01	12783301
CHCHD3	Early Stages	True	CHCHD3	0.33	18422935
CHES1	Early Stages	False	FOXN3	0.33	18422935
CHRM3	US	True	CHRM3	0.01	31747477
CHST7	US	True	CHST7	0.64	24734033
CHUK	US	True	CHUK	0.01	20940109
CIB1	US	True	CIB1	0.01	24741631
CKLF	Late Stages	True	CKLF	0.32	27913155
CLC	US	True	CLC	0.64	23958878

CLCP1	Early Stages	True	CLCP1	0.33	16128947
CLDN14	US	True	CLDN14	0.64	26842849
CLDN4	US	True	CLDN4	0.64	23958878
CLEC16A	US	True	CLEC16A	0.64	25057949;22961000;22257840;21399635;21399635;22961000;21399635;26394269
CLECSF6	US	False	CLEC4A	0.64	23958878
CLIC2	US	True	CLIC2	0.64	26267705
CLU	Early Stages	True	CLU	0.33	18422935
CNGB1	US	True	CNGB1	0.01	31033124
CNP	Early Stages	True	CNP	0.33	18422935
CNR1	Early Stages	True	CNR1	0.33	16128947
CNTN5	US	True	CNTN5	0.1	23000144
COG6	US	True	COG6	0.01	26993500
COL11A2	US	True	COL11A2	0.64	32363322
COL17A1	US	True	COL17A1	0.1	23000144
COL1A1	US	True	COL1A1	0.2	20056896
COL3A1	Late Stages	True	COL3A1	0.32	27913155
COL3A2	Late Stages	False	#N/A	0.32	27913155
COL6A2	US	True	COL6A2	0.64	16128947;31948396
COL6A3	Late Stages	True	COL6A3	0.32	16128947
COL9A3	US	True	COL9A3	0.64	23958878;21169553
COLCA2	US	True	COLCA2	0.64	29297981
CORO2B	Early Stages	True	CORO2B	0.33	18422935
CPEB1	US	True	CPEB1	0.2	26627607
CREB	Late Stages	False	#N/A	0.32	11488641
CREM	US	True	CREM	0.64	26084578
CRHR1	US	True	CRHR1	0.64	25057949
CRLA4	US	False	#N/A	0.64	29159718
CRYAA	Early Stages	True	CRYAA	0.33	18422935
CRYAB	Early Stages	True	CRYAB	0.33	18422935
CSE1L	Early Stages	True	CSE1L	0.33	18422935
CSF1R	US	True	CSF1R	0.64	23958878
CSNK2A2	US	True	CSNK2A2	0.64	30259846
CSPG4	Early Stages	True	CSPG4	0.33	18422935
CST7	US	True	CST7	0.64	23958878
CTAG1A	US	True	CTAG1A	0.64	24734033
CTAG1B	US	True	CTAG1B	0.64	24734033
CTHRC1	Late Stages	True	CTHRC1	0.32	31102333
CTLA4	US	True	CTLA4	0.64	24734033;18049163;31027870;19333938;25942345;22609442;21594562;21722820;10782900;18778710;23432218;15378793;20557968;22118691;174825

					23;22414241;18930330;12911663;28922436;28642883
CTLA-4	US	False	CTLA4	0.64	16034472
CTLA-4+49A/G	US	False	#N/A	0.64	16034472
CTNNA2	Early Stages	True	CTNNA2	0.33	16128947
CTNNBIP1	US	True	CTNNBIP1	0.64	29266316
CTSL	US	True	CTSL	0.64	23958878
CTSW	US	True	CTSW	0.64	23958878
CTSZ	US	True	CTSZ	0.02	29795304;30087368
CX3CR1	US	True	CX3CR1	0.02	21636249;15726664
CXCL10	Early Stages	True	CXCL10	0.33	23480180;16243485;11207658;18422935
CXCL11	US	True	CXCL11	0.64	32363322
CXCL12	US	True	CXCL12	0.01	29414663
CXCL3	US	True	CXCL3	0.64	32363322
CXCL8	US	True	CXCL8	0.64	32363322;21731723;27860118;17448566;26644386
CXCL9	Early Stages	True	CXCL9	0.33	16243485;18422935
CXCR3	Late Stages	True	CXCR3	0.32	27913155;23480180;16243485
CXCR4	Late Stages	True	CXCR4	0.32	27913155
CXCR5	US	True	CXCR5	0.64	26000122;21399635;26084578
CXCR6	US	True	CXCR6	0.64	19333938
CYGB	US	True	CYGB	0.3	30026087
CYP21A2	US	True	CYP21A2	0.64	32363322;30854688
CYP27B1	US	True	CYP27B1	0.64	32363322
CYP2D6	Late Stages	True	CYP2D6	0.32	16034472;15690482
CYP2E1	US	True	CYP2E1	0.64	16034472
CYP3A4	US	True	CYP3A4	0.02	17696930;16584389
CYP4B1	Early Stages	True	CYP4B1	0.33	16128947
CYP7A1	Early Stages	True	CYP7A1	0.33	28570655;23354620;17696930;20857261
DAG1	US	True	DAG1	0.2	12177244
DAP3	Early Stages	True	DAP3	0.33	18422935
DBT	US	True	DBT	0.03	8617422;9873825;14599987
DCN	Early Stages	True	DCN	0.33	18422935

DCTN4	US	True	DCTN4	0.02	30623621;28333129
DCTN6	US	True	DCTN6	0.01	15300582
DCTPP1	Late Stages	True	DCTPP1	0.32	11488641
DDR1	US	True	DDR1	0.01	21356365
DDR2	US	True	DDR2	0.01	12765478
DDX11	Late Stages	True	DDX11	0.32	11488641
DDX41	US	True	DDX41	0.64	24734033
DDX58	Late Stages	True	DDX58	0.32	27913155
DDX6	US	True	DDX6	0.64	22961000
DEFB1	US	True	DEFB1	0.01	15382127
DEFB4A	US	True	DEFB4A	0.01	15382127
DEFB4B	US	True	DEFB4B	0.01	15382127
DELEC1	US	True	DELEC1	0.1	23000144;28425483
DENND1B	US	True	DENND1B	0.64	26084578;22961000;22961000;21399635;26394269;21399635
DES	Late Stages	True	DES	0.32	28889961
DGCR2	US	True	DGCR2	0.01	21169553
DGKQ	US	True	DGKQ	0.64	29159718;26394269
DGKQ	US	True	DGKQ	0.64	29159718;26394269
DHH	US	True	DHH	0.64	23392275;25057949
DHX9	Early Stages	True	DHX9	0.33	18422935
DLAT	Early Stages	True	DLAT	0.33	18422935;8460168;9566826;9407346;20180236;29127360;9802945;29540861;10441182;21735469;17360156;28062665;16278592;11804659;14568249;15120760;8102256;8133065;12935787;14672611;28187915;19638108;30344796;30155449;14599987;8565296;9089911;22178199;12360422;10102567;12108679;12395322;28406960;31664722;27784538;30721

					553;30343708;23352659;7521314;18644699;12447863;11050037;0385636;985260
DLEU1	US	True	DLEU1	0.1	26394269
DLST	US	True	DLST	0.04	12753811;14599987;10385636;10441183
DMBT1	US	True	DMBT1	0.01	12368192
DMGDH	Late Stages	True	DMGDH	0.32	11488641
DNAAF2	Late Stages	True	DNAAF2	0.32	11488641
DNASE1	US	True	DNASE1	0.01	28263100
DNER	US	True	DNER	0.64	11488641
DNMT1	US	True	DNMT1	0.64	29297981
DNMT3A	US	True	DNMT3A	0.64	34033851
DPB1	US	False	#N/A	0.64	17931190
DPEP3	US	True	DPEP3	0.64	34033851
DPP8	US	True	DPP8	0.01	23704821
DPP9	US	True	DPP9	0.01	23704821
DPT	Early Stages	True	DPT	0.33	18422935
DR3	US	False	TNFRSF25	0.64	16034472
DR5	US	False	TNFRSF10B	0.64	7904771
DRB4101	US	False	#N/A	0.64	8738955
DRD2	Late Stages	True	DRD2	0.32	16128947
DSP	US	True	DSP	0.64	23958878
DUSP1	Early Stages	True	DUSP1	0.33	18422935
DUSP2	US	True	DUSP2	0.64	23958878
DUSP6	Early Stages	True	DUSP6	0.33	27913155
DYNC1LI1	Late Stages	True	DYNC1LI1	0.32	16128947
DYNLL1	Late Stages	True	DYNLL1	0.32	16128947
E2D2	US	FALSE	#N/A	0.64	31948396
E2F1	Late Stages	True	E2F1	0.32	24619556
E2F3	Early Stages	True	E2F3	0.33	26267705
EBAG9	US	True	EBAG9	0.01	12044529
EBI3	Early Stages	True	EBI3	0.33	29445068
ECSIT	Early Stages	True	ECSIT	0.33	27913155
EDN-11	US	False	#N/A	0.64	10756087
EDN3	Early Stages	True	EDN3	0.33	16128947
EGLN1	Late Stages	True	EGLN1	0.32	11488641
EGR2	US	True	EGR2	0.64	32363322
EIF4E	US	True	EIF4E	0.1	23000144;23000144
ELF1	US	True	ELF1	0.64	26084578
ELF2	Early Stages	True	ELF2	0.33	18422935
ELMO1	US	True	ELMO1	0.64	29159718;22961000;22961000;21399635;21399635

EMB	Early Stages	True	EMB	0.33	18422935
ENOS	US	False	NOS3	0.64	16034472
ENTPD2	US	True	ENTPD2	0.3	15651265
ESR1	US	True	ESR1	0.02	12169981;26608979
ESR2	US	True	ESR2	0.02	26608979;28013213
EST	Late Stages	False	MAP3K8 /// SULT1E1	0.32	11488641
ESTP1L1	Early Stages	False	#N/A	0.33	18422935
ETS1	US	True	ETS1	0.64	29159718;34033851
ETS-1	US	False	ETS1	0.64	31873148
EXOC3L4	US	True	EXOC3L4	0.1	26394269;21399635
FADD	Late Stages	True	FADD	0.32	16128947
FAM104B	US	True	FAM104B	0.64	24734033
FAM14B	Early Stages	False	IFI27L1	0.33	18422935
FAM155A	US	True	FAM155A	0.64	19458352
FAM177A1	US	True	FAM177A1	0.64	34033851
FAM35A	Early Stages	False	SHLD2	0.33	18422935
FANCG	Early Stages	True	FANCG	0.33	16128947
FAS	Late Stages	True	FAS	0.32	15929764
FASLG	US	True	FASLG	0.64	32363322;11394648
FBL	US	True	FBL	0.02	29533114;30583236
FBLN5	Early Stages	True	FBLN5	0.33	18422935
FBXL20	US	True	FBXL20	0.1	21399635;21399635
FCER2	Late Stages	True	FCER2	0.32	27913155
FCGR2B	Early Stages	True	FCGR2B	0.33	27913155
FCGR3A	Late Stages	True	FCGR3A	0.32	23958878
FCN1	US	True	FCN1	0.64	23958878
FCN2	Early Stages	True	FCN2	0.33	18422935
FCRL3	US	True	FCRL3	0.64	21299530
FDPS	Late Stages	True	FDPS	0.32	11488641
FER1L3	US	False	MYOF	0.64	23958878
FEZ1	Early Stages	True	FEZ1	0.33	27913155
FGD1	US	True	FGD1	0.64	24734033
FGF19	Late Stages	True	FGF19	0.32	27696157;28570655;30900136;29968724;26293907
FGFR4	Late Stages	True	FGFR4	0.32	26293907
FGG	Early Stages	True	FGG	0.33	18422935
FGL1	Early Stages	True	FGL1	0.33	18422935
FH	US	True	FH	0.01	18355017
FKBP4	Early Stages	True	FKBP4	0.33	16128947
FN1	Late Stages	True	FN1	0.32	30559459
FOS	Early Stages	True	FOS	0.33	18422935
FOSL1	Late Stages	True	FOSL1	0.32	11559656
FOXF1	US	True	FOXF1	0.64	22936693

FOXO1	US	True	FOXO1	0.01	24741631
FOXO3	US	True	FOXO3	0.01	23295054
FOXP3	US	True	FOXP3	0.64	25701076;17518371;28146070;17158635;19302244;16126962
FST	Early Stages	True	FST	0.33	18422935
FUNDC2	Early Stages	True	FUNDC2	0.33	26150899
FXR	Late Stages	False	NR1H4	0.32	26293907
FXYD6	Early Stages	True	FXYD6	0.33	18422935
FYN	US	True	FYN	0.64	23958878
GABPA	US	True	GABPA	0.02	20055754;28333129
GAGE12B	US	True	GAGE12B	0.64	24734033
GARS	Early Stages	False	GARS1	0.33	18422935
GATM	US	True	GATM	0.01	21594562
GCKR	US	True	GCKR	0.64	26084578
GGT1	US	True	GGT1	0.03	29193593;31021038;23910013
GGT2	US	True	GGT2	0.03	23910013;29193593;31021038
GGTLC1	US	True	GGTLC1	0.02	27755231;26644386
GGTLC3	US	True	GGTLC3	0.03	29193593;31021038;23910013
GLB1	US	True	GLB1	0.01	18536059
GLDC	Early Stages	True	GLDC	0.33	16128947
GLI3	US	True	GLI3	0.1	23000144
GLRX	Late Stages	True	GLRX	0.32	11488641
GNLY	US	True	GNLY	0.01	18584314
GOLM1	US	True	GOLM1	0.64	29297981
GOLPH2	US	False	GOLM1	0.64	29297981
GPBAR1	US	True	GPBAR1	0.02	28844960; 30357770
GPCR5A	Early Stages	False	#N/A	0.33	18422935
GPNMB	Late Stages	True	GPNMB	0.32	30559459
GPR65	US	True	GPR65	0.64	26084578
GPT	US	True	GPT	0.04	26644386;31807995;29889683;28263100
GRAMD1C	Early Stages	True	GRAMD1C	0.33	18422935
GRB2	Late Stages	True	GRB2	0.32	16128947
GRIK1	US	True	GRIK1	0.1	23000144
GRIPAP1	US	True	GRIPAP1	0.64	33675743
GSDMB	US	True	GSDMB	0.64	22936693;34033851;28588209;22936693; 22961000
GSDMC	US	True	GSDMC	0.64	28255561
GSDML	Early Stages	False	GSDMB	0.33	18422935

GSTM1	US	True	GSTM1	0.01	8491405
GSTM2	US	True	GSTM2	0.01	8491405
GSTP1	Late Stages	True	GSTP1	0.32	16128947
GTF2H1	US	True	GTF2H1	0.02	30623621;28333129
GTPBP6	US	True	GTPBP6	0.64	24734033
GYPA	Early Stages	True	GYPA	0.33	18422935
GZF1	Late Stages	True	GZF1	0.32	16128947
GZMA	US	True	GZMA	0.64	32363322
GZMB	US	True	GZMB	0.01	29429758
H3P10	US	True	H3P10	0.02	11171828;31812332
H3P23	US	True	H3P23	0.01	15300582
H63	Early Stages	False	CASC4	0.33	18422935
HAMP	US	True	HAMP	0.21	23704825;17515961
HAO1	Early Stages	True	HAO1	0.33	18422935
HAO2	Late Stages	True	HAO2	0.32	11488641
HAP1	US	True	HAP1	0.01	18671305
HCCS	US	True	HCCS	0.64	24734033
HCK	Late Stages	True	HCK	0.32	27913155
HCP5	US	True	HCP5	0.64	32363322
HDAC9	US	True	HDAC9	0.01	27915160
HEXA	Late Stages	True	HEXA	0.32	11488641
HGF	US	True	HGF	0.03	16941151;16859527;7806142
HGFAC	Early Stages	True	HGFAC	0.33	16128947
HHIP	US	True	HHIP	0.2	18375471
HIF1A	US	True	HIF1A	0.31	22271822;22271822
HIG1	Early Stages	False	HIGD1A	0.33	18422935
HK1	US	True	HK1	0.04	31001269;9468341;29554146;27784538
HMGB1	US	True	HMGB1	0.01	29774570
HMOX1	US	True	HMOX1	0.2	12114196
HMOX2	Late Stages	True	HMOX2	0.32	16128947
HNF4A	Late Stages	True	HNF4A	0.32	23354620;17696930; 23354620
HOXA4	Early Stages	True	HOXA4	0.33	16128947
HOXD3	Early Stages	True	HOXD3	0.33	16128947
HOXD4	US	True	HOXD4	0.64	24734033
HPGDS	US	True	HPGDS	0.01	8491405
HPR	US	True	HPR	0.64	23958878
HSD11B1	Early Stages	True	HSD11B1	0.33	27913155
HSPA5BP1	Early Stages	False	TMEM132A	0.33	18422935
HSPCA	Early Stages	False	HSP90AA1 /// HSP90AA2P	0.33	18422935
HSPD1	US	True	HSPD1	0.02	15120760;7526135

HSPG2	Early Stages	True	HSPG2	0.33	18422935
ICAM1	Late Stages	True	ICAM1	0.32	27913155;9089909;16038038;7904546
ICAM-1	US	False	#N/A	0.64	29297981;34033851
ICAM3	Early Stages	True	ICAM3	0.33	18422935
ICOS	US	True	ICOS	0.64	32363322;19333938
ICOSLG	US	True	ICOSLG	0.64	26084578
ID2	US	True	ID2	0.64	31948396
IDH3G	US	True	IDH3G	0.64	24734033
IDS	US	True	IDS	0.64	24734033
IFI16	Late Stages	True	IFI16	0.32	27913155
IFI27	US	True	IFI27	0.01	15300582
IFI44L	US	True	IFI44L	0.64	24734033
IFIT1	US	True	IFIT1	0.64	24734033
IFNG	US	True	IFNG	0.09	31803181;17518371;29445068;16128947;8706330;9096578;16243485;8076766;30119881
IFN-GAMMA	Early Stages	False	#N/A	0.33	10824891
IFT80	US	True	IFT80	0.64	22936693;34033851
IGF1	US	True	IGF1	0.01	15256976
IGF2	US	True	IGF2	0.64	26267705
IGFBP1	Early Stages	True	IGFBP1	0.33	18422935
IGHG3	US	True	IGHG3	0.01	12791319
IKZF3	US	True	IKZF3	0.64	28588209;30643196;20639880;20639880;23000144;22936693;22961000;26394269;28425483;28062665;23000144;23000144
IL-1	Early Stages	False	#N/A	0.33	16034472
IL10	US	True	IL10	0.1	17518371;15880308;20638239;9625317;30274824;17158635;15884119;12765479;10634216;31555703
IL-10	Late Stages	False	IL10	0.32	31255417
IL11RA	Early Stages	True	IL11RA	0.33	18422935
IL12A	US	True	IL12A	0.64	28425483;30119881;20639880;19458352;

					24648611;27175695;22961000
IL12B	US	True	IL12B	0.64	26084578;20639880
IL12RB	US	False	IL12RB1	0.64	26000122
IL12RB1	US	True	IL12RB1	0.52	28299343;23910013;24648611
IL12RB2	US	True	IL12RB2	0.64	20639880;23152861;19458352;22961000;20639880;21399635;26394269;30119881;21399635;28299343;22936693;19458352;19458352;22961000
IL13	US	True	IL13	0.64	24734033;17518371
IL13RA2	Early Stages	True	IL13RA2	0.33	27913155
IL16	US	True	IL16	0.64	28425483;28425483
IL17A	US	True	IL17A	0.64	24734033;19101114;19604266;31555703;29414663;23910013
IL18	Late Stages	True	IL18	0.32	27913155;20303781
IL18R1	US	True	IL18R1	0.01	25327457
IL1A	US	True	IL1A	0.01	8706330
IL1B	US	True	IL1B	0.64	8076766;19604266;31231399;8706330
IL1RL1	US	True	IL1RL1	0.64	29159718
IL1RL2	US	True	IL1RL2	0.64	29159718
IL1RN	US	True	IL1RN	0.64	23958878;12169981
IL2	US	True	IL2	0.03	10634216;15880308;26347073
IL21	US	True	IL21	0.64	28425483;21304239;28425483
IL21R	US	True	IL21R	0.64	30259846;28425483;28425483
IL23A	US	True	IL23A	0.01	23910013
IL-23R	US	False	IL23R	0.64	26000122
IL27	US	True	IL27	0.64	26084578
IL2RA	US	True	IL2RA	0.02	20650610;17518371

IL32	Early Stages	True	IL32	0.33	18422935
IL37	US	True	IL37	0.01	24481870
IL4	US	True	IL4	0.64	24734033;20442198;8565296;9096578;10634216;29414663
IL4R	US	True	IL4R	0.64	28425483
IL5	US	True	IL5	0.03	10634216;7806143;17518371
IL6	US	True	IL6	0.64	24734033;15884119
IL-6	US	False	IL6	0.64	32363322
IL6R	US	True	IL6R	0.64	31948396
IL7	Early Stages	True	IL7	0.33	29266628;29429758
IL-7	US	False	IL7 /// LINC02605	0.64	29429758
IL7R	US	True	IL7R	0.64	26084578;28062665;21399635;22961000;21399635
IL8	Late Stages	False	CXCL8	0.32	32363322
ILF3	Late Stages	True	ILF3	0.32	27913155
IMPDH2	Early Stages	True	IMPDH2	0.33	18422935
C1orf106	US	True	INAVA	0.64	34033851
INTU	US	True	INTU	0.01	31254667
IP6K3	US	True	IP6K3	0.64	32363322
IRAK1	US	True	IRAK1	0.64	24734033
IRF2	Late Stages	True	IRF2	0.32	27913155
IRF4	Late Stages	True	IRF4	0.32	27913155
IRF5	US	True	IRF5	0.64	21506939;21506939;22961000;22961000
IRF7	US	True	IRF7	0.64	34033851
IRF8	US	True	IRF8	0.64	29159718
IRS2	Early Stages	True	IRS2	0.33	18422935
ISG20	Late Stages	True	ISG20	0.32	27913155;17518371
ITCH	Early Stages	True	ITCH	0.33	27913155
ITGA1	US	True	ITGA1	0.01	31803181
ITGA5	US	True	ITGA5	0.01	21116829
ITGA6	Late Stages	True	ITGA6	0.32	27913155
ITGAL	US	True	ITGAL	0.01	16038038
ITGAV	US	True	ITGAV	0.01	21116829
ITGAX	Late Stages	True	ITGAX	0.32	27913155
ITGB1	Late Stages	True	ITGB1	0.32	27913155
ITGB2	Late Stages	True	ITGB2	0.32	27913155;16038038
ITGB6	Late Stages	True	ITGB6	0.32	18538673
ITGB8	US	True	ITGB8	0.64	34033851
ITIH4	US	True	ITIH4	0.01	31737133
ITLN1	US	True	ITLN1	0.01	29550924

JAK2	US	True	JAK2	0.64	26084578;24619965
JAM3	US	True	JAM3	0.01	29753567
JMJD1C	Early Stages	True	JMJD1C	0.33	18422935
JPH3	US	True	JPH3	0.01	8937759
KANSL1	US	True	KANSL1	0.1	22961000
KBTBD6	US	True	KBTBD6	0.64	24734033
KCND1	US	True	KCND1	0.64	33675743
KCNMA1	US	True	KCNMA1	0.64	32363322
KEAP1	US	True	KEAP1	0.31	28333129;30026087
KHDRBS1	US	True	KHDRBS1	0.02	30623621;28333129
KIAA0379	Early Stages	False	ANKRD28	0.33	18422935
KIAA0888	Early Stages	False	FAM169A	0.33	18422935
KIAA0922	Early Stages	False	TMEM131L	0.33	18422935
KIAA0977	Early Stages	False	COBLL1	0.33	18422935
KIAA1683	US	False	IQCN	0.64	22936693
KIAA1754	US	False	ITPRIP	0.64	23958878
KIAA1815	Early Stages	False	ERMP1	0.33	18422935
KLF10	Early Stages	True	KLF10	0.33	18422935
KLF4	US	True	KLF4	0.01	31549733
KLHL1	US	True	KLHL1	0.02	14568249;12763363
KLHL12	US	True	KLHL12	0.02	31001269;29554146
KLRC1	Late Stages	True	KLRC1	0.32	27913155
KLRG1	US	True	KLRG1	0.01	31255417
KMT2B	US	True	KMT2B	0.01	31549733
KMT2D	US	True	KMT2D	0.01	31549733
KRT18	Late Stages	True	KRT18	0.32	29380060
KRT20	US	True	KRT20	0.02	24744588;30450101
KRT7	US	True	KRT7	0.3	21681009
L1CAM	US	True	L1CAM	0.01	29510897
LACC1	US	True	LACC1	0.64	26084578
LAIR2	Late Stages	True	LAIR2	0.32	27913155
LAMA4	US	True	LAMA4	0.64	29297981;34033851
LAMP1	US	True	LAMP1	0.01	22098537
LAMP2	Late Stages	True	LAMP2	0.32	27913155
LARP6	Early Stages	True	LARP6	0.33	18422935
LBP	Early Stages	True	LBP	0.33	27913155
LCP2	Late Stages	True	LCP2	0.32	16128947
LDB2	Early Stages	True	LDB2	0.33	18422935
LEF1	US	True	LEF1	0.64	32363322
LEP	US	True	LEP	0.01	30307540
LGALS1	Early Stages	True	LGALS1	0.33	18422935
LGALS3	US	True	LGALS3	0.01	31231399
LGALS3BP	US	True	LGALS3BP	0.02	31724755;29318378
LILRB1	Late Stages	True	LILRB1	0.32	27913155
LILRB2	US	True	LILRB2	0.64	23958878
LILRB3	US	True	LILRB3	0.64	23958878

LIN28B	Early Stages	True	LIN28B	0.33	26267705
LMO2	US	True	LMO2	0.64	23958878
LORICRIN	US	True	LORICRIN	0.01	16023247
LOX	US	True	LOX	0.31	16023247;16023247
LOXL2	US	True	LOXL2	0.31	16023247;16023247
LPA	US	True	LPA	0.01	8937759
LRG1	US	True	LRG1	0.01	30471232
LRP1	Early Stages	True	LRP1	0.33	29550924
LRRC17	Early Stages	True	LRRC17	0.33	16128947
LRRC32	US	True	LRRC32	0.01	31033124
LRRFIP2	Early Stages	True	LRRFIP2	0.33	18422935
LSM4	Early Stages	True	LSM4	0.33	18422935
LTA	US	True	LTA	0.01	1684248
LTB	US	True	LTB	0.64	32363322
LTBR	US	True	LTBR	0.64	22961000
LTF	US	True	LTF	0.64	32363322
LXRA	Early Stages	False	NR1H3	0.33	19669248
LY6G5B	US	True	LY6G5B	0.64	32363322
LY86	Late Stages	True	LY86	0.32	27913155
C5orf30	US	False	#N/A	0.64	29159718
MAGEA3	US	True	MAGEA3	0.64	24734033
MAGEA6	US	True	MAGEA6	0.64	24734033
MAGEA9	US	True	MAGEA9	0.64	24734033
MAGED4B	US	True	MAGED4B	0.64	24734033
MAL	US	True	MAL	0.64	31948396
MANBA	US	True	MANBA	0.64	26394269;21399635;22961000
MAP2K1	Early Stages	True	MAP2K1	0.33	27913155
MAP3K1	Late Stages	True	MAP3K1	0.32	27913155
MAP3K14	US	True	MAP3K14	0.2	12810685;9620319;17060023;11884426;16640660;17986312;9010048;14764671;10910194
MAP3K7IP1	US	False	TAB1	0.64	26084578
MAP4	Early Stages	True	MAP4	0.33	18422935
MAPK14	US	True	MAPK14	0.3	30026087
MAPK3	Late Stages	True	MAPK3	0.32	27913155
MAPK4	Early Stages	True	MAPK4	0.33	16128947
MAPT	US	True	MAPT	0.64	22961000;22961000
MARCKSL1	US	True	MARCKSL1	0.01	12763363
MARCO	Early Stages	True	MARCO	0.33	27913155
MAS1	US	True	MAS1	0.2	17532087
MAVS	Early Stages	True	MAVS	0.33	27913155
MBL2	US	False	MBL2 /// MBL3P	0.64	11712863
MBP	Early Stages	True	MBP	0.33	18422935

MCAM	Late Stages	True	MCAM	0.32	27913155
MCART1	Early Stages	False	SLC25A51	0.33	18422935
MCM6	Early Stages	True	MCM6	0.33	18422935
MCPIP1	Late Stages	False	ZC3H12A	0.32	33513427
MDK	Early Stages	True	MDK	0.33	18422935
MDR1	US	False	ABCB1 /// TBC1D9	0.64	16034472
MED1	US	True	MED1	0.1	21399635
MED13	Late Stages	True	MED13	0.32	11488641
MEF2C	Early Stages	True	MEF2C	0.33	18422935
MFGE8	Late Stages	True	MFGE8	0.32	27913155
MFSD6	Early Stages	True	MFSD6	0.33	18422935
MGC35366	Early Stages	False	AMDHD1	0.33	18422935
MGLL	US	True	MGLL	0.64	23958878
MGST3	US	True	MGST3	0.64	29297981
MICA	US	True	MICA	0.64	27913155
MIF	Early Stages	True	MIF	0.33	27913155;23913513
MIHB	US	False	BIRC2	0.64	31948396
MME	Early Stages	True	MME	0.33	27913155
MMEL1	US	True	MMEL1	0.64	26000122;20639879
MMP1	Late Stages	True	MMP1	0.32	11488641
MMP10	Late Stages	True	MMP10	0.32	11806373
MMP13	US	True	MMP13	0.2	20056896
MMP2	US	True	MMP2	0.64	32363322
MMP3	US	True	MMP3	0.2	20056896
MMP9	US	True	MMP9	0.2	20056896
MOCOS	US	True	MOCOS	0.64	23958878
MRP2	US	False	ABCC2 /// KLHL1 /// SYCE1L	0.64	18662272
MRP3	US	False	ABCC3	0.64	18662272
MRP4	Late Stages	False	ABCC4	0.32	17696930
MS4A1	US	True	MS4A1	0.02	30450101;24744588
MS4A2	Early Stages	True	MS4A2	0.33	16128947
MTARC2	Early Stages	True	MTARC2	0.33	18422935
MTCP1	US	True	MTCP1	0.64	24734033
MTM1	US	True	MTM1	0.64	24734033
MTMR3	US	True	MTMR3	0.64	26084578
MTMR8	US	True	MTMR8	0.64	24734033
MTTFA	Early Stages	False	#N/A	0.33	15652467
MTTP	Early Stages	True	MTTP	0.33	19669248
MUC16	US	True	MUC16	0.64	29297981;34033851
MUTED	Early Stages	False	BLOC1S5	0.33	18422935
MYB	Early Stages	True	MYB	0.33	26267705
MYBPC3	Early Stages	True	MYBPC3	0.33	16128947
MYCL1	Early Stages	False	MYCL	0.33	18422935
MYD88	US	True	MYD88	0.01	17448566
MYO1F	US	True	MYO1F	0.64	29297981;34033851

NAB1	US	True	NAB1	0.1	26394269;21399635;21399635
NADSYN1	US	True	NADSYN1	0.64	22936693;34033851
NAT10	US	True	NAT10	0.03	29193593;31714880;14687829
NBPF3	US	True	NBPF3	0.01	21691115
NDFIP1	US	True	NDFIP1	0.64	34033851
NEAT1	Early Stages	True	NEAT1	0.33	18422935
NELFCD	US	True	NELFCD	0.02	30087368;29795304
NEO1	Late Stages	True	NEO1	0.32	16128947
NF-ATC	US	False	NFATC1	0.64	31948396
NFE2	Early Stages	True	NFE2	0.33	16128947
NFE2L1	Early Stages	True	NFE2L1	0.33	15652467
NFE2L2	Late Stages	True	NFE2L2	0.32	30026087;20055754;28333129
NFKB1	US	True	NFKB1	0.64	26084578;21399635;28425483;23000144;28062665
NFKB2	Late Stages	True	NFKB2	0.32	27913155
NFYA	US	True	NFYA	0.01	18671305
NGFR	US	True	NGFR	0.64	31948396
NHS	US	True	NHS	0.64	24734033
NKFB1	US	False	#N/A	0.64	26000122
NKG7	US	True	NKG7	0.64	23958878
NKX2-3	US	True	NKX2-3	0.64	26084578
NLRC5	Late Stages	True	NLRC5	0.32	27913155
NME3	US	True	NME3	0.64	31948396
NNMT	Late Stages	True	NNMT	0.32	11488641
NOLA3	Early Stages	False	NOP10	0.33	18422935
NOS1	Late Stages	True	NOS1	0.32	16128947
NOS2	US	True	NOS2	0.5	21903766;30026087
NOS3	US	True	NOS3	0.31	12974901;30026087
NOTCH1	US	True	NOTCH1	0.64	19458352;29248458
NOTCH2	Late Stages	True	NOTCH2	0.32	11488641
NQO1	US	True	NQO1	0.01	16610002
NR0B2	US	True	NR0B2	0.01	31549733
NR1H4	US	True	NR1H4	0.02	27696157;30928103
NR1I2	US	True	NR1I2	0.03	20416375;26504856;15690482
NR1I3	US	True	NR1I3	0.01	16550034
NR1L2	Early Stages	False	#N/A	0.33	18930330
NR3C1	US	True	NR3C1	0.01	15635817
NR4A2	US	True	NR4A2	0.64	23958878
NRAMP1	US	False	SLC11A1	0.64	16034472

NRAS	Early Stages	True	NRAS	0.33	30690835;28192189
NRDC	Late Stages	True	NRDC	0.32	11488641
NRF-1	Early Stages	False	#N/A	0.33	15652467
NRP1	Late Stages	True	NRP1	0.32	27913155
NS5ATP13TP2	Early Stages	False	#N/A	0.33	18422935
NSA2	US	True	NSA2	0.3	18422935
NSEP1	Early Stages	False	YBX1	0.33	18422935
NSF	US	True	NSF	0.1	22961000
NTN1	US	True	NTN1	0.64	29297981
NTRK2	US	True	NTRK2	0.64	29297981
NUP210	US	True	NUP210	0.07	21594562;31714880;31281353;28083929;30854688;31737133;22519587
NUP62	US	True	NUP62	0.02	30623621;28333129
OAS3	Late Stages	True	OAS3	0.32	27913155
OGDH	US	True	OGDH	0.04	8527402;11050037;8617422;10385636
OLFML3	Early Stages	True	OLFML3	0.33	18422935
OLIG3	US	True	OLIG3	0.64	28255561
OPRM1	US	True	OPRM1	0.01	18709298
ORC1L	US	False	ORC1	0.64	24734033
ORMDL3	US	True	ORMDL3	0.64	22936693;34033851;22961000;28588209;22961000;28588209
OTUD5	US	True	OTUD5	0.64	33675743
OVGP1	Early Stages	True	OVGP1	0.33	18422935
PAM	US	True	PAM	0.64	30259846
PAX3	US	True	PAX3	0.1	23000144
PBX1	US	True	PBX1	0.64	29297981;34033851
PCDH7	US	True	PCDH7	0.64	19458352
PDC	US	True	PDC	0.03	11343230;11050037;8565296
PDCD1	US	True	PDCD1	0.01	18041714
PDCD1LG2	US	True	PDCD1LG2	0.01	17311651
PDE5A	US	True	PDE5A	0.2	17610866
PDGFB	Early Stages	True	PDGFB	0.33	25690649;25327457
PDGFRA	US	True	PDGFRA	0.64	31948396
PDHX	US	True	PDHX	0.03	10385636;10385634;11050037
PDIR	US	False	PDIA2 /// PDIA5	0.64	31948396

PDK4	US	True	PDK4	0.01	24293610
PDLIM3	US	True	PDLIM3	0.03	14687829;31714880;29193593
PDLIM4	Early Stages	True	PDLIM4	0.33	18422935
PDP1	US	True	PDP1	0.01	1911885
PDZD2	US	True	PDZD2	0.01	17518371
PDZD4	US	True	PDZD4	0.64	24734033
PECAM1	US	True	PECAM1	0.64	23958878
PER1	US	True	PER1	0.64	23958878
PHF16	US	False	JADE3	0.64	24734033
PIF1	Early Stages	True	PIF1	0.33	18422935
PIG7	US	False	LITAF	0.64	31948396
PIK3CA	US	True	PIK3CA	0.01	29482551
PIM2	US	True	PIM2	0.64	33675743
PIN1	US	True	PIN1	0.64	29297981;34033851
PIN4	US	True	PIN4	0.64	26267705
PKC	Early Stages	False	PRRT2	0.33	16128947
PLAUR	US	True	PLAUR	0.64	32363322
PLCB1	US	True	PLCB1	0.1	23000144
PLCL1	US	True	PLCL1	0.64	26084578
PLCL2	US	True	PLCL2	0.64	26084578;21399635;26394269;21399635
PLEKHC1	US	False	FERMT2	0.64	23958878
PLIZF	Early Stages	False	#N/A	0.33	16128947
PLS3	US	True	PLS3	0.64	23958878
PM5	Late Stages	False	NOMO1 /// NOMO2	0.32	11488641
PML	US	True	PML	0.06	8131741;22503841;8986606;7631159;9089912;9230084
PNMA1	Late Stages	True	PNMA1	0.32	27913155
PNPLA3	US	True	PNPLA3	0.01	25297933
PNRC1	US	True	PNRC1	0.64	23958878
POGLUT1	US	True	POGLUT1	0.64	30643196
POLA1	US	True	POLA1	0.64	31948396
POLD	US	False	POLD1	0.64	22936693
POLR2G	US	True	POLR2G	0.01	28056976
POU2AF1	US	True	POU2AF1	0.64	26084578;28062665;23000144;23000144;23000144;23000144;28062665
POU2F1	Early Stages	True	POU2F1	0.02	23612856;24620780
POU5F1	US	True	POU5F1	0.64	32363322
PPA1	Late Stages	True	PPA1	0.32	11488641
PPARA	US	True	PPARA	0.64	30100231;31077509;31312410;30928103
PPARG	Late Stages	True	PPARG	0.32	15880426

PPARG2	Early Stages	False	PPARG	0.33	16128947
PPARGC1A	Late Stages	True	PPARGC1A	0.32	23354620;24293610
PPIH	Early Stages	True	PPIH	0.33	18422935
PPP1R10	US	True	PPP1R10	0.64	32363322
PPP1R16B	US	True	PPP1R16B	0.64	23958878
PPP1R18	US	True	PPP1R18	0.64	32363322
PPP1R2C	US	True	PPP1R2C	0.01	9468341
PPP2R1B	Early Stages	True	PPP2R1B	0.33	18422935
PQBP1	US	True	PQBP1	0.64	33675743
PRAM1	US	True	PRAM1	0.01	8131741
PRDM1	US	True	PRDM1	0.64	32363322;34033851
PRDM16	US	True	PRDM16	0.1	23000144
PRDX5	US	True	PRDX5	0.64	26084578
PRELP	Early Stages	True	PRELP	0.33	18422935
PRF1	US	True	PRF1	0.64	23958878
PRICKLE1	US	True	PRICKLE1	0.1	21399635
PRKAR2B	US	True	PRKAR2B	0.64	23958878
PRKCB	US	True	PRKCB	0.64	29159718;28062665;28062665
PRKCD	Late Stages	True	PRKCD	0.32	27913155
PRKCE	Late Stages	True	PRKCE	0.32	27913155
PRKX	US	True	PRKX	0.64	24734033
PRM1	US	True	PRM1	0.64	30992889
PRM2	US	True	PRM2	0.64	30992889
PRO2061	Late Stages	False	#N/A	0.32	11488641
PROSTASIN	Late Stages	False	#N/A	0.32	11488641
PRPF38A	US	True	PRPF38A	0.64	24734033
PRPF40A	US	True	PRPF40A	0.01	11712863
PRR3	US	True	PRR3	0.64	32363322
PRRC2A	US	True	PRRC2A	0.01	1672123
PSD4	US	True	PSD4	0.64	23958878
PSEN1	Late Stages	True	PSEN1	0.32	27913155
PSMA4	US	True	PSMA4	0.01	15866219
PSMB9	US	True	PSMB9	0.64	32363322
PSMD3	US	True	PSMD3	0.1	22936693
PSMD4	Late Stages	True	PSMD4	0.32	11488641
PSMD9	US	True	PSMD9	0.01	15300582
PSMG4	US	True	PSMG4	0.1	28425483
PTGS2	US	True	PTGS2	0.01	11584358
PTPN22	US	True	PTPN22	0.64	26000122;16671954;28922436;27406031
PVT1	US	True	PVT1	0.64	22936693;34033851
PXR	US	False	NR1I2	0.64	16034472
RAB6	Late Stages	False	RAB6A	0.32	16128947
RAC1	Early Stages	True	RAC1	0.33	18422935
RAD51B	US	True	RAD51B	0.64	26394269;22961000;21399635;22961000;21399635

RAD51C	Late Stages	True	RAD51C	0.32	22936693;34033851;30259846
RAD51L1	US	False	RAD51B	0.64	22936693;34033851
RAN	Early Stages	True	RAN	0.33	18422935
RANKL	US	False	TNFSF11	0.64	30259846
RARB	Late Stages	True	RARB	0.32	34033851
RASGRP2	US	True	RASGRP2	0.64	23958878
RASGRP3	Early Stages	True	RASGRP3	0.33	18422935
RASGRP4	US	True	RASGRP4	0.64	23958878
RBBP4	Early Stages	True	RBBP4	0.33	18422935
RBFOX1	US	True	RBFOX1	0.01	20153395
RBM45	US	True	RBM45	0.1	23543758;26473500;7699227;21563204;15713222;18416479;19003916;7904771;23809616;16481299;19018986;21953406;28588884
RBP1	Early Stages	True	RBP1	0.33	18422935
RBP4	Late Stages	True	RBP4	0.32	11488641
RDX	US	True	RDX	0.01	14568249
REL	US	True	REL	0.64	26084578
RELA	Early Stages	True	RELA	0.33	27913155;30026087
RELB	Late Stages	True	RELB	0.32	27913155
REN	US	True	REN	0.2	22266601
REPS1	Early Stages	True	REPS1	0.33	27913155
RGPR	Early Stages	False	SEC16B	0.33	18422935
RGS1	Late Stages	True	RGS1	0.32	16128947
RHOA	Late Stages	True	RHOA	0.32	16128947
RHOBTB3	Early Stages	True	RHOBTB3	0.33	18422935
RIBC1	US	True	RIBC1	0.64	24734033
RIN3	US	True	RIN3	0.64	34033851
RIPK3	US	True	RIPK3	0.01	30144553
RMI2	US	True	RMI2	0.64	22936693
RNASET2	US	True	RNASET2	0.01	28922436
RND3	Late Stages	True	RND3	0.32	16128947
RNF11	Late Stages	True	RNF11	0.32	11488641
RNF128	US	True	RNF128	0.64	24734033
RORC	Early Stages	True	RORC	0.33	27913155
RPE	US	True	RPE	0.64	23958878
RPL10	Early Stages	True	RPL10	0.33	18422935
RPL27	Early Stages	True	RPL27	0.33	18422935
RPL3	US	True	RPL3	0.64	21399635
RPL35A	Early Stages	True	RPL35A	0.33	18422935
RPL37A	Late Stages	True	RPL37A	0.32	11488641
RPL39L	Early Stages	True	RPL39L	0.33	18422935
RPL9	Early Stages	True	RPL9	0.33	18422935
RPLP2	Early Stages	True	RPLP2	0.33	18422935
RPS13	Early Stages	True	RPS13	0.33	18422935

RPS2	Late Stages	True	RPS2	0.32	18422935
RPS27L	Early Stages	True	RPS27L	0.33	18422935
RPS28	Early Stages	True	RPS28	0.33	18422935
RPS6KA4	US	True	RPS6KA4	0.64	29159718;21399635;21399635
RPS6KB1	US	True	RPS6KB1	0.2	15769867
RRAGD	Early Stages	True	RRAGD	0.33	18422935
RUNX1	Late Stages	True	RUNX1	0.32	27913155
RUNX3	US	True	RUNX3	0.64	26084578
RXRC	US	False	#N/A	0.64	29297981;34033851
RXRG	US	True	RXRG	0.64	29297981;34033851
S100A12	US	True	S100A12	0.64	23958878;25313445
S100A4	Early Stages	True	S100A4	0.33	18422935
S100A8	Late Stages	True	S100A8	0.32	16128947
S100A9	Late Stages	True	S100A9	0.32	16128947
S100B	Late Stages	True	S100B	0.32	27913155
S100P	Early Stages	True	S100P	0.33	18422935
SAT1	Late Stages	True	SAT1	0.32	11488641
SCHIP1	US	True	SCHIP1	0.64	27175695
SCLY	US	True	SCLY	0.64	24734033
SCNN1A	US	True	SCNN1A	0.64	30992889
SCRIB	Late Stages	True	SCRIB	0.32	11488641
SCTR	US	True	SCTR	0.01	31251081
SDC1	Late Stages	True	SDC1	0.32	11488641
SDF2	US	True	CXCL12	0.64	31948396
SDK1	US	True	SDK1	0.1	23000144
SERPIEST4	Early Stages	False	#N/A	0.33	18422935
SERPINA1	US	True	SERPINA1	0.01	1080923
SERPINB2	US	True	SERPINB2	0.64	23958878
SERPINB3	US	True	SERPINB3	0.01	16549823
SERPING1S	Early Stages	False	#N/A	0.33	18422935
SF3B1	Early Stages	True	SF3B1	0.33	18422935
SGPL1	Late Stages	True	SGPL1	0.32	11488641
SH2B3	US	True	SH2B3	0.64	29159718;22961000
SH2D1A	US	True	SH2D1A	0.64	32363322
SHH	US	True	SHH	0.64	23392275;25057949
SHPS-1	Late Stages	False	SIRPA	0.32	11488641
SHROOM4	US	True	SHROOM4	0.64	24734033
SIAE	US	True	SIAE	0.01	22257840
SKIL	Late Stages	True	SKIL	0.32	16128947
SKIV2L	US	True	SKIV2L	0.1	30854688
SLAMF1	Late Stages	True	SLAMF1	0.32	27913155
SLAMF6	US	True	SLAMF6	0.64	32363322
SLC10A1	US	True	SLC10A1	0.02	14568249;12763363
SLC10A2	US	True	SLC10A2	0.02	29704003;28468009
SLC10A3	US	True	SLC10A3	0.64	24734033
SLC11A1	US	True	SLC11A1	0.64	23958878

SLC12A9	US	True	SLC12A9	0.03	18456456;22098537;18536059
SLC17A4	US	True	SLC17A4	0.64	32363322
SLC17A8	US	True	SLC17A8	0.1	23000144
SLC22A1	US	True	SLC22A1	0.02	24620780;23612856
SLC22A23	US	True	SLC22A23	0.1	28425483
SLC25A1	US	True	SLC25A1	0.01	27995739
SLC25A5	Early Stages	True	SLC25A5	0.33	18422935
SLC35A1	Late Stages	True	SLC35A1	0.32	11488641
SLC35A2	US	True	SLC35A2	0.64	33675743
SLC44A1	US	True	SLC44A1	0.01	24620780
SLC4A2	US	True	SLC4A2	0.39	29540861;19491853;18930330;28962898;21594562;21691115;18188457;12029638;22383162;27592379
SLC51A	US	True	SLC51A	0.31	16423920;29420067
SLC51B	US	True	SLC51B	0.3	16423920
SLC52A3	US	True	SLC52A3	0.64	19458352
SLC9A6	US	True	SLC9A6	0.64	24734033
SLCO1B1	US	True	SLCO1B1	0.02	14568249;12763363
SLCO1B3	US	True	SLCO1B3	0.01	14568249
SLCO1C1	US	True	SLCO1C1	0.2	15770136
SLIT2	US	True	SLIT2	0.01	31545977
SLITRK2	US	True	SLITRK2	0.64	24734033
SLITRK4	US	True	SLITRK4	0.64	24734033
SLP-76	Late Stages	False	LCP2	0.32	16128947
SLPI	US	True	SLPI	0.03	14687829;31714880;29193593
SMAD2	US	True	SMAD2	0.01	15566516
SMAD3	US	True	SMAD3	0.01	15566516
SMARCA1	US	True	SMARCA1	0.64	24734033
SNRPC	Early Stages	True	SNRPC	0.33	16128947
SOAT1	US	True	SOAT1	0.01	26394269
SOCS1	US	True	SOCS1	0.64	27913155;22257840;28146070
SOCS3	US	True	SOCS3	0.64	32363322
SOD2	Early Stages	True	SOD2	0.33	18422935
SOST	US	True	SOST	0.01	27019303
SP100	US	True	SP100	0.1	10052683;30854688;22503841;26347895;31281353;31714880;9230084;8986606;9089912;763115

					9;31111679;2 2519587
SP140L	US	True	SP140L	0.01	26347895
SPARC	Late Stages	True	SPARC	0.32	30559459
SPATA31A3	US	True	SPATA31A3	0.01	31033124
SPEF2	US	True	SPEF2	0.64	30992889
SPIB	US	True	SPIB	0.64	26000122;213 99635;206398 80;20639880; 22257840;206 39880;213996 35;26394269; 21506939
SPINT1	US	True	SPINT1	0.01	16941151
SPN	Late Stages	True	SPN	0.32	27913155
SPP1	US	True	SPP1	0.64	12558864
SPPL2C	US	True	SPPL2C	0.1	22961000
SPRY4	Early Stages	True	SPRY4	0.33	18422935
SQSTM1	US	True	SQSTM1	0.04	22098537;306 23621;283331 29;28249264
SREBP2	Early Stages	False	SREBF2	0.33	19669248
SRY	US	True	SRY	0.01	29248458
SS18L1	US	True	SS18L1	0.01	10347101
SSBP1	Early Stages	True	SSBP1	0.33	18422935
SSR4	US	True	SSR4	0.64	24734033
ST6GAL1	Early Stages	True	ST6GAL1	0.33	27913155
ST8SIA4	US	True	ST8SIA4	0.64	34033851
STAT1	US	True	STAT1	0.64	27913155;229 36693
STAT3	US	True	STAT3	0.64	12547716
STAT4	US	True	STAT4	0.64	26000122;318 03181;260845 78;30119881; 26347073;230 00144;229610 00;25798064; 23000144;229 36693;230001 44;28425483; 21399635;229 61000;246486 11
STAT5A	US	True	STAT5A	0.01	29429758
STAT5B	US	True	STAT5B	0.01	29429758
STH	US	True	STH	0.1	22961000
STK3	Early Stages	True	STK3	0.33	18422935
STX3A	Early Stages	False	STX3	0.33	18422935
SUI1	Early Stages	False	EIF1	0.33	18422935
SULT2A1	US	True	SULT2A1	0.02	26504856;176 96930

SYCE1L	US	True	SYCE1L	0.02	12763363;14568249
SYK	Late Stages	True	SYK	0.32	27913155
SYNGR1	US	True	SYNGR1	0.64	29159718;22936693;26993500;22961000;26394269;22961000
SYT17	Early Stages	True	SYT17	0.33	27913155
TAB1	US	True	TAB1	0.64	26676814
TAB2	US	True	TAB2	0.64	12547716
TAF9	Early Stages	True	TAF9	0.33	24734033
TAF9B	US	True	TAF9B	0.64	24734033
TAP1	Late Stages	True	TAP1	0.32	27913155
TARS	Early Stages	False	TARS1	0.33	18422935
TARS1	Late Stages	True	TARS1	0.32	11488641
TBC1D1	US	True	TBC1D1	0.64	26084578
TBC1D9	US	True	MDR1	0.03	16034472
TBX21	US	True	TBX21	0.01	30119881
TCEAL6	US	True	TCEAL6	0.64	24734033
TDGF1	Late Stages	True	TDGF1	0.32	16128947
TEP1	Late Stages	True	TEP1	0.32	15690482
TET2	US	True	TET2	0.64	34033851
TFF3	US	True	TFF3	0.01	12395334
TFIIB	US	False	GTF2B	0.64	31948396
TGFB1	US	True	TGFB1	0.32	30026087;17911416;17158635
TGFBR2	US	True	TGFBR2	0.02	25057952;29375127
TGM2	US	True	TGM2	0.01	12560760
THY1	Early Stages	True	THY1	0.33	18422935
TICAM2	US	True	TICAM2	0.01	15300582
TIMM17B	US	True	TIMM17B	0.64	33675743
TIMMDC1	US	True	TIMMDC1	0.64	26394269;23000144;22961000;23000144;21399635;28062665;22961000;22936693;30643196;21399635
TJAM_HUMAN	US	False	#N/A	0.64	23958878
TLR10	US	True	TLR10	0.64	32363322
TLR2	US	True	TLR2	0.02	20940109;15856047
TLR3	US	True	TLR3	0.01	15856047
TLR4	US	True	TLR4	0.06	20940109;31115023;16629651;17448566;16006099;23142582
TLR6	Late Stages	True	TLR6	0.32	27913155
TLR7	US	True	TLR7	0.64	32363322

TLR8	US	True	TLR8	0.64	32363322
TLR9	Early Stages	True	TLR9	0.33	16034472;15878652
TMED7	Late Stages	True	TMED7	0.32	15300582;15300582
TMED7-TICAM2	US	True	TMED7-TICAM2	0.01	15300582
TMEM163	US	True	TMEM163	0.64	34033851
TMEM39A	US	True	TMEM39A	0.64	22936693;21399635;22961000;28425483
TMEM60	US	True	TMEM60	0.64	23958878
TNF	US	True	TNF	0.64	10365808;16938647;10068101;23011034;15856047;10515837;19758199;8706330;1684248;10453936;12911663;20578265;26644386
TNFA	US	False	TNF	0.64	18930330
TNFAIP2	US	True	TNFAIP2	0.64	26084578
TNFAIP3	US	True	TNFAIP3	0.64	30259846
TNFAIP8L2	US	True	TNFAIP8L2	0.01	26644386
TNFR17	Early Stages	False	#N/A	0.33	16128947
TNFRSF11A	US	True	TNFRSF11A	0.01	31377883
TNFRSF11B	Late Stages	True	TNFRSF11B	0.32	27913155;19229472
TNFRSF12A	US	True	TNFRSF12A	0.64	23958878
TNFRSF17	Early Stages	True	TNFRSF17	0.33	16128947
TNFRSF1A	US	True	TNFRSF1A	0.64	28425483;21399635;21399635;26394269;22961000;22961000;21399635
TNFRSF6B	US	True	TNFRSF6B	0.64	26084578
TNFRSF7	US	False	CD27	0.64	16128947
TNFSF10	US	True	TNFSF10	0.01	18385934
TNFSF11	US	True	TNFSF11	0.64	19229472;22936693;31377883
TNFSF13B	US	True	TNFSF13B	0.01	27529417
TNFSF15	US	True	TNFSF15	0.64	27913155;30643196;30906791;23000144;26084578;29773854;28062665;28062665;24453414;275

					07062;23000144;23000144;25899471;23000144
TNFSF18	US	True	TNFSF18	0.64	26084578
TNFSF4	US	True	TNFSF4	0.01	23622253
TNFSF8	US	True	TNFSF8	0.64	23000144
TNFSF9	US	True	TNFSF9	0.01	20303781
TNNT1	US	True	TNNT1	0.64	23958878
TNP03	US	False	#N/A	0.64	21538281
TNP2	US	True	TNP2	0.64	22936693
TNPO3	US	True	TNPO3	0.64	26084578;21399635;22936693;21506939;22961000;21399635;22961000
TNSF4	US	False	#N/A	0.64	26000122
TNXB	US	True	TNXB	0.64	32363322
TOPBP1	Early Stages	True	TOPBP1	0.33	18422935
TP53	Late Stages	True	TP53	0.32	11394648;8302580
TP63	US	True	TP63	0.1	23000144
TPMT	US	True	TPMT	0.01	17241387
TPT1	Late Stages	True	TPT1	0.32	11488641
TRA	US	True	TRA	0.64	16034472
TRAF1	US	True	TRAF1	0.64	23710202
TRAF3	US	True	TRAF3	0.64	22936693
TRAF3IP2	US	True	TRAF3IP2	0.64	29297981
TRIM14	US	True	TRIM14	0.64	34033851
TRIM15	US	True	TRIM15	0.64	32363322
TRIM21	US	True	TRIM21	0.03	29554146;30581434;28447402
TRPC3	US	True	TRPC3	0.64	19458352
TSBP1	US	True	TSBP1	0.64	19458352;19458352
TST	Late Stages	True	TST	0.32	11488641
TTC38	Early Stages	True	TTC38	0.33	18422935
TTC39A	Late Stages	True	TTC39A	0.32	11488641
TTR	US	True	TTR	0.64	23958878
TUBA1A	Late Stages	True	TUBA1A	0.32	16128947
TUSC3	US	True	TUSC3	0.64	24734033
TYK2	US	True	TYK2	0.64	29159718;26394269;22961000;22961000;22961000
TYROBP	US	True	TYROBP	0.64	23958878
UBD	Early Stages	True	UBD	0.33	18422935
UBE2D1	US	True	UBE2D1	0.64	26084578
UBE2D3	US	True	UBE2D3	0.64	32363322
UBL4A	US	True	UBL4A	0.64	24734033
UGT2B4	US	True	UGT2B4	0.01	17696930
UGT3A1	US	True	UGT3A1	0.64	30992889

VAMP3	US	True	VAMP3	0.64	26084578
VCAM1	US	True	VCAM1	0.64	16938647
VCX2	US	True	VCX2	0.64	24734033
VCX3A	US	True	VCX3A	0.64	24734033
VDR	US	True	VDR	0.64	10611163;11230990;24224838;15880308;11786968;25730037;10877221;11230734;12169981;24526415;28146070;15683428;19376604
VEGFA	US	True	VEGFA	0.2	26627607
VEGFB	US	True	VEGFB	0.64	31948396
VIM	Late Stages	True	VIM	0.32	30559459
VPS53	Early Stages	True	VPS53	0.33	18422935
VRP	Late Stages	False	TBC1D8 /// VEGFC	0.32	11488641
VTCN1	US	True	VTCN1	0.01	21120594
VTN	Early Stages	True	VTN	0.33	18422935
WASF1	US	True	WASF1	0.64	10498630
WASHC2C	Late Stages	True	WASHC2C	0.32	11488641
WDFY4	US	True	WDFY4	0.64	34033851
WDR11	US	True	WDR11	0.64	24265779
WDR15	US	False	WDR11	0.64	26000122
WDR16	US	False	CFAP52	0.64	29297981;34033851
WDR33	Early Stages	True	WDR33	0.33	18422935
WDR5B	Early Stages	True	WDR5B	0.33	18422935
WDR70	Early Stages	True	WDR70	0.33	18422935
WDR8	US	False	WRAP73	0.64	23958878
WDRPUH	US	False	CFAP52	0.64	29297981;34033851
WISP3	US	False	CCN6	0.64	29297981;34033851
WLS	Late Stages	True	WLS	0.32	12763363
WNT2B	US	True	WNT2B	0.01	11488641
WNT3	US	True	WNT3	0.1	22961000
WNT5A	Early Stages	True	WNT5A	0.33	18422935
WRNIP1	Late Stages	True	WRNIP1	0.32	11488641
WSB1	Late Stages	True	WSB1	0.32	11488641
XCE	US	True	XCE	0.64	30343705
XPR1	US	True	XPR1	0.02	28808886;31041784
YDJC	US	True	YDJC	0.64	26084578
YIPF6	US	True	YIPF6	0.64	24734033
ZBTB16	Early Stages	True	ZBTB16	0.33	16128947
ZC3HAV1	US	True	ZC3HAV1	0.64	34033851
ZEB1	Early Stages	True	ZEB1	0.33	16128947
ZFP36	Early Stages	True	ZFP36	0.33	18422935
ZIC3	US	True	ZIC3	0.64	24734033
ZKSCAN3	US	True	ZKSCAN3	0.64	32363322
ZKSCAN4	US	True	ZKSCAN4	0.64	32363322

ZMAT1	US	True	ZMAT1	0.64	16128947
ZMIZ1	US	True	ZMIZ1	0.64	26084578
ZNF182	US	True	ZNF182	0.64	24734033
ZNF217	US	True	ZNF217	0.64	23958878
ZNF365	US	True	ZNF365	0.64	26084578
ZNF559	US	True	ZNF559	0.64	29297981;34033851
ZNRD2	US	True	ZNRD2	0.01	15300582
ZPBP2	US	True	ZPBP2	0.64	22936693;22961000;28588209
ZYX	Early Stages	True	ZYX	0.33	16128947

US: unspecified stages.

Table S2: Over-representation analysis (ORA) from Seed Genes results for drugs. Results are stratified by disease stages (Label column).

Label	geneSet	description	size	overlap	expect	enrichment Ratio	P Value	FDR
Early Stages	PA164712838	Interleukin inhibitors	260	14	2.765	5.063	7e-07	0.002
Early Stages	PA164712839	Interleukins	351	15	3.733	4.018	5.2e-06	0.008
Early Stages	PA448797	carbohydrates	790	23	8.403	2.737	1.06e-05	0.009
Early Stages	DB00054	Abciximab	14	4	0.149	26.862	1.14e-05	0.009
Early Stages	DB00075	Muromonab	15	4	0.16	25.072	1.54e-05	0.009
Early Stages	DB09130	Copper	177	10	1.883	5.312	1.97e-05	0.01
Early Stages	DB11638	Artenimol	84	7	0.893	7.835	3.08e-05	0.013

Early Stages	PA164712787	Heparins or heparinoids for topical use	210	10	2.234	4.477	8.41e-05	0.028
Early Stages	PA449855	heparin	210	10	2.234	4.477	8.41e-05	0.028
Early Stages	PA450197	l-leucine	451	15	4.797	3.127	9.56e-05	0.029
Late Stages	PA164712908	Monoclonal antibodies	348	26	3.431	7.579	<3.33e-16	<1.25e-14
Late Stages	PA164712817	Immunoglobulins	704	32	6.94	4.611	<3.33e-16	<1.25e-14
Late Stages	PA164754884	immune globulin	704	32	6.94	4.611	<3.33e-16	<1.25e-14
Late Stages	PA452611	epipodophyllotoxin	121	13	1.193	10.899	<1.94e-10	<1.17e-07
Late Stages	PA450993	podophyllotoxin	121	13	1.193	10.899	<3.33e-16	<1.25e-14
Late Stages	PA451999	interferons	342	19	3.371	5.636	<3.33e-16	<1.25e-14
Late Stages	PA164712447	Antiinfectives	529	23	5.215	4.41	<3.33e-16	<1.25e-14

Late Stages	PA164713366	Tumor necrosis factor alpha (TNF-alpha) inhibitors	177	14	1.745	8.024	<2.26e-09	<8.51e-07
Late Stages	PA452621	antineoplastic agents	492	22	4.85	4.536	<3.33e-16	<1.25e-14
Late Stages	PA452174	antivirals	418	20	4.121	4.854	<3.33e-16	<1.25e-14
US	PA164712817	Immunoglobulins	704	95	26.299	3.612	<3.33e-16	<1.25e-14
US	PA164754884	immune globulin	704	95	26.299	3.612	<3.33e-16	<1.25e-14
US	PA164712447	Antiinfectives	529	96	19.762	4.858	<3.33e-16	<1.25e-14
US	PA452621	antineoplastic agents	492	89	18.379	4.842	<3.33e-16	<1.25e-14
US	PA452174	antivirals	418	91	15.615	5.828	<3.33e-16	<1.25e-14
US	PA164712839	Interleukins	351	100	13.112	7.627	<3.33e-16	<1.25e-14
US	PA164712908	Monoclonal antibodies	348	59	13	4.538	<3.33e-16	<1.25e-14

US	PA164713235	Respiratory System	348	55	13	4.231	<3.33e-16	<1.25e-14
US	PA451999	interferons	342	90	12.776	7.044	<3.33e-16	<1.25e-14
US	PA164713267	Skeleton	328	49	12.253	3.999	<3.33e-16	<1.25e-14
US	PA164713274	Specific immunoglobulins	312	57	11.655	4.891	<3.33e-16	<1.25e-14
US	PA164712362	Analgesics	269	49	10.049	4.876	<3.33e-16	<1.25e-14
US	PA164713346	Thiazides, combinations with psycholeptics and/or analgesics	269	49	10.049	4.876	<3.33e-16	<1.25e-14
US	PA164712838	Interleukin inhibitors	260	74	9.713	7.619	<3.33e-16	<1.25e-14
US	PA449515	etanercept	41	13	1.53	8.487	1.62e-09	2.97e-08
US	PA450566	mycophenolate mofetil	30	9	1.12	8.030	9.43e-07	8.69e-06

US	PA10832	corticosteroids	204	31	7.620	4.067	2.58e-11	5.80e-10
US	PA164712819	Immunostimulants	236	61	8.816	6.919	<3.33e-16	<1.25e-14
US	PA164712966	Other Antineoplastic Agents	228	42	8.517	4.931	<3.33e-16	<1.25e-14
US	PA164712458	Antiinflammatory Agents	214	45	7.994	5.629	<3.33e-16	<1.25e-14
US	PA164713378	Vaccines	206	46	7.695	5.978	<3.33e-16	<1.25e-14
US	PA164712871	Liver therapy	177	41	6.612	6.201	<3.33e-16	<1.25e-14
US	PA164713366	Tumor necrosis factor alpha (TNF-alpha) inhibitors	177	47	6.612	7.108	<3.33e-16	<1.25e-14
US	PA10420	colony stimulating factors	148	35	5.529	6.331	<3.33e-16	<1.25e-14
US	PA130620649	granulocyte colony-stimulat	137	33	5.118	6.448	<3.33e-16	<1.25e-14

		ing factor						
US	PA164713093	Other immunostimulants	104	28	3.885	7.207	<3.33e-16	<1.25e-14
US	PA164712454	Antiinfectives For Systemic Use	96	32	3.586	8.923	<3.33e-16	<1.25e-14
US	PA164713273	Specific Antirheumatic Agents	92	30	3.437	8.729	<3.33e-16	<1.25e-14
US	PA164712504	Antivirals For Systemic Use	74	28	2.764	10.129	<3.33e-16	<1.25e-14
US	PA164712520	Bacterial Vaccines	52	21	1.943	10.811	<3.33e-16	<1.25e-14
US	PA164713047	Other antivirals	215	39	8.032	4.856	<3.33e-16	<1.25e-14
US	PA164713204	Protein kinase inhibitors	504	61	18.828	3.24	<3.33e-16	<1.25e-14
US	PA164712976	Other Drugs For Disorde	110	28	4.109	6.813	<3.33e-16	<1.25e-14

		rs Of The Musculo-skeletal System						
US	PA164712476	Antineovascularisation agents	315	47	11.767	3.994	<3.33e-16	<1.25e-14
US	PA164712976	Other Drugs For Disorders Of The Musculo-skeletal System	110	28	4.109	6.814	<3.33e-16	<1.25e-14
US	PA164713390	Viral Vaccines	147	31	5.491	5.645	<3.33e-16	<1.25e-14
US	DB04348	Taurocholic Acid	27	15	1.009	14.872	<3.77e-15	<1.33e-13
US	PA164712327	Alimentary Tract And Metabolism	424	53	15.839	3.346	<3.33e-16	<1.25e-14
US	PA164712557	Biguanides	62	9	2.316	3.885	4.61e-5	2.16e-4

US	PA164712600	Cardiovascular System	591	64	22.078	2.899	<3.33e-16	<1.25e-14
US	PA449107	collagenase	138	29	5.155	5.625	<3.33e-16	<1.25e-14
US	PA164712732	Enzyme inhibitors	573	62	21.405	2.896	3.48e-14	1.10e-12
US	PA164712789	Hepatitis vaccines	36	16	1.345	11.897	<3.33e-16	<1.25e-14
US	PA10799	anakinra	39	16	1.457	10.982	1.98e-13	5.96e-13
US	PA451363	simvastatin	97	14	3.623	3.863	1.41e-05	1.03e-4
US	DB01076	atorvastatin	23	6	0.859	6.983	1.55e-4	8.61e-4
US	PA164712882	Macrolides	134	13	5.006	2.597	1.52e-3	5.99e-3

US: unspecified-stages.

Table S3: Over-representation analysis (ORA) from Likely Positives (LP) results for drugs. Results are stratified by disease stages (Label column).

Label	geneSet	description	size	overlap	expect	Enrichment Ratio	P Value	FDR
Early Stages	PA450280	l-lysine	932	45	8.462287	5.317712	<3.33e-16	<1.25e-14

Early Stages	PA451673	l-threonine	875	40	7.944743	5.034776	<3.33e-16	<1.25e-14
Early Stages	PA164713204	Protein kinase inhibitors	504	34	4.576172	7.42979	<3.33e-16	<1.25e-14
Early Stages	PA452621	antineoplastic agents	492	32	4.467216	7.163298	<3.33e-16	<1.25e-14
Early Stages	PA164712476	Antineovascularisation agents	315	22	2.860108	7.692018	9.35E-14	5.63E-11
Early Stages	PA164712966	Other Antineoplastic Agents	228	19	2.070173	9.177976	2.24E-13	1.12E-10
Early Stages	PA164713176	Platinum compounds	180	17	1.634347	10.40171	5.81E-13	2.50E-10
Early Stages	PA451330	l-serine	993	35	9.016149	3.881923	1.98E-12	7.45E-10
Early Stages	PA449014	cisplatin	234	18	2.124651	8.471978	3.91E-12	1.31E-09
Early Stages	PA164712732	Enzyme inhibitors	573	25	5.202672	4.805223	5.79E-11	1.74E-08
Late Stages	PA451673	l-threonine	875	36	6.355795	5.664122	<3.33e-16	<1.25e-14
Late Stages	PA164713204	Protein kinase inhibitors	504	38	3.660938	10.37985	<3.33e-16	<1.25e-14
Late Stages	PA452621	antineoplastic agents	492	29	3.573773	8.114674	<3.33e-16	<1.25e-14
Late Stages	PA134687924	erlotinib	48	11	0.348661	31.54929	3.24E-14	2.33E-11
Late Stages	PA164712732	Enzyme inhibitors	573	26	4.162138	6.24679	3.86E-14	2.33E-11
Late Stages	PA153561371	EGFR inhibitors	78	12	0.566574	21.17995	3.44E-13	1.46E-10
Late Stages	PA450280	l-lysine	932	31	6.769829	4.57914	3.80E-13	1.46E-10
Late Stages	PA165109623	staurosporine	127	14	0.922498	15.17618	3.87E-13	1.46E-10
Late Stages	PA152031327	geldanamycin	66	11	0.479409	22.94494	1.38E-12	4.63E-10

Late Stages	PA451330	l-serine	993	31	7.212919	4.297844	2.01E-12	6.06E-10
US	PA451330	l-serine	993	45	8.887347	5.063378	<3.33e-16	<1.25e-14
US	PA450280	l-lysine	932	52	8.341397	6.233968	<3.33e-16	<1.25e-14
US	PA451673	l-threonine	875	50	7.831247	6.384679	<3.33e-16	<1.25e-14
US	PA448372	amino acids	770	40	6.891498	5.804254	<3.33e-16	<1.25e-14
US	PA452621	antineoplastic agents	492	31	4.403398	7.040017	<3.33e-16	<1.25e-14
US	PA152031327	geldanamycin	66	16	0.5907	27.08652	<3.33e-16	0
US	PA165109623	staurosporine	127	17	1.13665	14.95624	1.33E-15	4.46E-13
US	PA164713206	Proteolytic enzymes	806	35	7.213697	4.851881	2.66E-15	8.02E-13
US	PA165291490	tanespimycin	30	10	0.2685	37.24396	6.12E-14	1.67E-11
US	PA451822	l-tyrosine	700	31	6.264998	4.948126	8.39E-14	2.11E-11
US	PA164713066	Other cytotoxic antibiotics	96	14	0.8592	16.29423	1.39E-13	3.21E-11
US	PA153561371	EGFR inhibitors	78	11	2.91381	2.745546	8.532e-3	2.54e-2
US	PA164712966	Other Antineoplastic Agents	228	16	2.040599	7.840834	2.09E-10	4.20E-08
US	PA152241909	PP2	66	10	0.5907	16.92907	3.27E-10	6.15E-08
US	PA134687924	erlotinib	48	9	0.4296	20.94973	3.53E-10	6.26E-08
US	PA165109660	genistein	53	9	0.47435	18.97334	8.99E-10	1.50E-07
US	PA164712386	Anti-estrogens	124	12	1.1098	10.81276	1.10E-09	1.75E-07
US	DB00997	Doxorubicin	31	11	1.15805	9.498711	7.71e-09	1.29E-07
US	PA449552	etoposide	161	13	1.440949	9.021829	2.06E-09	3.10E-07
US	DB00675	Tamoxifen	38	8	1.41954	5.635599	6.55e-05	4.06E-04
US	PA450632	nilutamide	8	5	0.0716	69.83243	2.93E-09	4.20E-07
US	PA450197	l-leucine	451	20	4.036449	4.954851	3.48E-09	4.76E-07
US	DB12695	Phenethyl Isothiocyanate	44	8	0.3938	20.31489	4.53E-09	5.94E-07
US	PA451249	rifabutin	30	7	0.2685	26.07077	6.77E-09	8.49E-07

US	PA164713176	Platinum compounds	180	13	1.610999	8.069525	8.03E-09	9.67E-07
US	PA452611	epipodophyllotoxin	121	11	1.08295	10.15744	1.09E-08	1.20E-06
US	PA450993	podophyllotoxin	121	11	1.08295	10.15744	1.09E-08	1.20E-06
US	PA450636	nitrile	94	10	0.8413	11.88637	1.13E-08	1.20E-06
US	PA164712838	Interleukin inhibitors	260	15	2.326999	6.44607	1.16E-08	1.20E-06
US	PA451159	protease inhibitors	348	17	3.114599	5.458167	1.40E-08	1.40E-06
US	PA164743471	adenosine triphosphate	350	17	3.132499	5.426977	1.52E-08	1.48E-06
US	PA164712476	Antineovascularisation agents	315	16	2.819249	5.67527	2.23E-08	2.06E-06
US	PA164712349	Amides	234	14	2.094299	6.684814	2.32E-08	2.06E-06
US	PA449014	cisplatin	234	14	2.094299	6.684814	2.32E-08	2.06E-06
US	PA164712750	Ethers	104	10	0.9308	10.74345	3.03E-08	2.61E-06
US	PA151958596	curcumin	60	8	0.537	14.89758	5.81E-08	4.86E-06
US	PA164713366	Tumor necrosis factor alpha (TNF-alpha) inhibitors	177	12	1.584149	7.575043	6.26E-08	5.09E-06
US	PA164712406	Antibiotics	217	13	1.942149	6.693615	7.44E-08	5.90E-06
US	PA7000	sorafenib	42	7	0.3759	18.62198	8.20E-08	6.33E-06

US: unspecified-stages.

Table S4: Over-representation analysis (ORA) from Seed genes results for pathways. Results are stratified by disease stages (Label column).

**Label**	**geneSet**	**description**	**size**	**overlap**	**expect**	**Enrichment Ratio**	**pValue**	**FDR**
Early Stages	R-HSA-168256	Immune System	1997	56	19.204	2.916	<3.33e-16	<1.25e-14
Early Stages	R-HSA-168249	Innate Immune System	1053	37	10.126	3.654	<3.33e-16	<1.25e-14

Early Stages	R-HSA-166658	Complement cascade	58	10	0.558	17.929	<3.33e-16	<1.25e-14
Early Stages	WP2806	Human Complement System	99	12	0.952	12.605	<3.33e-16	<1.25e-14
Early Stages	R-HSA-977606	Regulation of Complement cascade	47	9	0.452	19.913	<3.33e-16	0
Early Stages	R-HSA-168898	Toll-like Receptor Cascades	155	13	1.491	8.722	<3.33e-16	<1.25e-14
Early Stages	R-HSA-166665	Terminal pathway of complement	8	5	0.077	64.993	<3.33e-16	<1.25e-14
Early Stages	hsa04610	Complement and coagulation cascades	79	10	0.76	13.163	<3.33e-16	<1.25e-14
Early Stages	R-HSA-449147	Signaling by Interleukins	462	20	4.443	4.502	<3.33e-16	<1.25e-14
Early Stages	WP545	Complement Activation	22	6	0.212	28.361	<3.33e-16	<1.25e-14
Late Stages	R-HSA-168256	Immune System	1997	70	17.24	4.06	<3.33e-16	<1.25e-14
Late Stages	R-HSA-168249	Innate Immune System	1053	43	9.09	4.73	<3.33e-16	<1.25e-14

Late Stages	PA164712908	Monoclonal antibodies	348	26	3.004	8.654	<3.33e-16	<1.25e-14
Late Stages	R-HSA-1280215	Cytokine Signaling in Immune system	688	33	5.939	5.556	<3.33e-16	<1.25e-14
Late Stages	hsa05169	Epstein-Barr virus infection	201	20	1.735	11.526	6.66e-16	7.60e-13
Late Stages	hsa05200	Pathways in cancer	526	29	4.541	6.386	<3.33e-16	<1.25e-14
Late Stages	PA164712817	Immunoglobulins	704	32	6.078	5.265	<3.33e-16	<1.25e-14
Late Stages	PA164754884	immune globulin	704	32	6.078	5.265	<3.33e-16	<1.25e-14
Late Stages	WP4754	IL-18 signaling pathway	279	20	2.409	8.304	<3.33e-16	<1.25e-14
Late Stages	R-HSA-449147	Signaling by Interleukins	462	24	3.988	6.017	<3.33e-16	<1.25e-14
US	R-HSA-168256	Immune System	1997	203	83.253	2.438	<3.33e-16	<1.25e-14
US	R-HSA-1280215	Cytokine Signaling in Immune system	688	114	28.682	3.975	<3.33e-16	<1.25e-14

US	hsa05200	Pathways in cancer	526	77	21.928	3.511	<3.33e-16	<1.25e-14
US	R-HSA-449147	Signaling by Interleukins	462	84	19.26	4.361	<3.33e-16	<1.25e-14
US	hsa04060	Cytokine-cytokine receptor interaction	294	73	12.257	5.956	<3.33e-16	<1.25e-14
US	hsa05162	Measles	132	34	5.503	6.179	<3.33e-16	<1.25e-14
US	hsa04659	Th17 cell differentiation	107	30	4.461	6.725	<3.33e-16	<1.25e-14
US	hsa04620	Toll-like receptor signaling pathway	104	31	4.336	7.15	<3.33e-16	<1.25e-14
US	hsa04064	NF-kappa B signaling pathway	95	32	3.96	8.08	<3.33e-16	<1.25e-14
US	hsa05321	Inflammatory bowel disease (IBD)	65	31	2.71	11.44	<3.33e-16	<1.25e-14

US: unspecified-stages.

Table S5: Over-representation analysis (ORA) from Likely Positives (LP) results for pathways. Results are stratified by disease stages (Label column).

Label	geneSet	description	size	overlap	expect	enrichmentRatio	pValue	FDR
Early Stages	R-HSA-5663202	Diseases of signal transduction	378	30	3.998	7.505	<3.33e-16	<1.25e-14
Early Stages	hsa05215	Prostate cancer	97	18	1.026	17.547	<3.33e-16	<1.25e-14
Early Stages	R-HSA-597592	Post-translational protein modification	1425	51	15.07	3.384	<3.33e-16	<1.25e-14
Early Stages	hsa05200	Pathways in cancer	526	30	5.563	5.393	<3.33e-16	<1.25e-14
Early Stages	WP1984	Integrated Breast Cancer Pathway	155	18	1.639	10.981	<3.33e-16	<1.25e-14
Early Stages	hsa04120	Ubiquitin mediated proteolysis	137	17	1.449	11.733	<3.33e-16	<1.25e-14
Early Stages	R-HSA-212436	Generic Transcription Pathway	1169	42	12.363	3.397	<3.33e-16	<1.25e-14
Early Stages	R-HSA-3108232	SUMO E3 ligases SUMOylate target proteins	181	18	1.914	9.404	<3.33e-16	<1.25e-14

Early Stages	WP138	Androgen receptor signaling pathway	91	14	0.962	14.547	<3.33e-16	<1.25e-14
Early Stages	WP366	TGF-beta Signaling Pathway	133	16	1.407	11.375	<3.33e-16	<1.25e-14
Late Stages	hsa05150	Staphylococcus aureus infection	55	23	1.663	13.826	<3.33e-16	<1.25e-14
Late Stages	hsa05140	Leishmaniasis	73	15	2.208	6.794	<3.33e-16	<1.25e-14
Late Stages	hsa04610	Complement and coagulation cascades	79	15	2.389	6.278	<3.33e-16	<1.25e-14
Late Stages	hsa05322	Systemic lupus erythematosus	131	19	3.962	4.795	<3.33e-16	<1.25e-14
Late Stages	hsa05323	Rheumatoid arthritis	88	14	2.662	5.26	3e-07	<1.25e-14
Late Stages	hsa04145	Phagosome	151	18	4.567	3.941	6e-07	<1.25e-14
Late Stages	hsa05133	Pertussis	74	12	2.238	5.362	1.8e-06	<1.25e-14

Late Stages	hsa05310	Asthma	30	8	0.907	8.817	2e-06	<1.25e-14
Late Stages	hsa05152	Tuberculosis	176	18	5.323	3.381	5.4e-06	<1.25e-14
Late Stages	hsa05416	Viral myocarditis	58	10	1.754	5.701	7.6e-06	<1.25e-14
Late Stages	hsa05321	Inflammatory bowel disease (IBD)	64	10	1.936	5.166	1.89e-05	0.001
Late Stages	hsa04640	Hematopoietic cell lineage	96	12	2.904	4.133	2.88e-05	0.001
Late Stages	hsa04620	Toll-like receptor signaling pathway	101	12	3.055	3.928	4.82e-05	0.001
Late Stages	hsa05143	African trypanosomiasis	35	7	1.059	6.613	6.87e-05	0.002
Late Stages	hsa05144	Malaria	49	8	1.482	5.398	9.54e-05	0.002
Late Stages	hsa05330	Allograft rejection	37	7	1.119	6.255	1e-04	0.002
Late Stages	hsa05205	Proteoglycans in cancer	201	17	6.079	2.796	0.0001149	0.002
Late Stages	hsa05164	Influenza A	165	15	4.99	3.006	0.000131	0.002

Late Stages	hsa05332	Graft-versus-host disease	39	7	1.18	5.934	0.0001418	0.002
Late Stages	hsa04512	ECM-receptor interaction	82	10	2.48	4.032	0.0001661	0.003
Late Stages	hsa04940	Type I diabetes mellitus	41	7	1.24	5.645	0.0001969	0.003
Late Stages	hsa04659	Th17 cell differentiation	105	11	3.176	3.464	0.0003091	0.005
Late Stages	hsa04672	Intestinal immune network for IgA production	47	7	1.422	4.924	0.0004723	0.007
Late Stages	hsa05020	Prion diseases	35	6	1.059	5.668	0.0005566	0.008
Late Stages	hsa05320	Autoimmune thyroid disease	51	7	1.542	4.538	0.0007848	0.01
Late Stages	hsa04510	Focal adhesion	198	15	5.989	2.505	0.000933	0.012
Late Stages	hsa05168	Herpes simplex infection	180	14	5.444	2.572	0.0010665	0.013
Late Stages	hsa04514	Cell adhesion molecules (CAMs)	142	12	4.295	2.794	0.0011821	0.014

Late Stages	hsa05219	Bladder cancer	41	6	1.24	4.839	0.0013255	0.015
Late Stages	hsa04658	Th1 and Th2 cell differentiation	89	9	2.692	3.343	0.0013991	0.015
Late Stages	hsa04612	Antigen processing and presentation	73	8	2.208	3.623	0.0015266	0.016
Late Stages	hsa04657	IL-17 signaling pathway	91	9	2.752	3.27	0.0016376	0.017
Late Stages	hsa05146	Amoebiasis	96	9	2.904	3.1	0.0023784	0.023
Late Stages	hsa04933	AGE-RAGE signaling pathway in diabetic complications	98	9	2.964	3.036	0.0027405	0.026
US	hsa05200	Pathways in cancer	526	40	5.482	7.296	<3.33e-16	<1.25e-14
US	hsa04151	PI3K-Akt signaling pathway	354	28	3.689	7.589	<3.33e-16	<1.25e-14
US	hsa05169	Epstein-Barr virus infection	201	23	2.095	10.979	<3.33e-16	<1.25e-14

US	hsa05203	Viral carcinogenesis	201	26	2.095	12.411	<3.33e-16	<1.25e-14
US	hsa05167	Kaposi sarcoma-associated herpesvirus infection	186	28	1.939	14.444	<3.33e-16	<1.25e-14
US	hsa05161	Hepatitis B	144	26	1.501	17.324	<3.33e-16	<1.25e-14
US	hsa04210	Apoptosis	136	20	1.417	14.11	<3.33e-16	<1.25e-14
US	hsa05160	Hepatitis C	131	21	1.365	15.381	<3.33e-16	<1.25e-14
US	hsa04722	Neurotrophin signaling pathway	119	19	1.24	15.32	<3.33e-16	<1.25e-14
US	hsa04662	B cell receptor signaling pathway	71	16	0.74	21.622	<3.33e-16	<1.25e-14

US: unspecified-stages.

Supplementary File S1: Detailed Methodology.

Supplementary File S2: l-threonine Webgestalt results.

Supplementary File S3: l-serine Webgestalt results.

Supplementary File S4: l-lysine Webgestalt results.

Supplementary File S5: PA45261 Webgestalt results.

Supplementary File S6: PA164712966 Webgestalt results.

Supplementary File S7: PA164712732 Webgestalt results.
