# Supplementary material for: Network Proximity-Based Drug Repurposing Strategy for Early and Late Stages of Primary Biliary Cholangitis
Source: Biomedicines. 2022 Jul 13;10(7):1694. doi: 10.3390/biomedicines10071694 (PMC9312896; doi:10.3390/biomedicines10071694)
Supplement: Supplementary file 1 [file biomedicines-10-01694-s001.zip › Supplementary files S1 to S7/Supplementary File S1.pdf]

Supplementary material (file 1) to "Network Proximity-Based Drug Repurposing Strategy for Early and Late Stages of Primary Biliary Cholangitis"

E. Shaini[1], G. Pasculli[2,3], A. Mastropietro[3], P. Stolfi[4], P. Tieri[4], D. Vergni[4], R. Cozzolongo[1], F. Pesce[2] and G. Giannelli[1]

[1]National Institute of Research "Saverio De Bellis", Castellana Grotte (BA), Italy;

[2]Department of Emergency and Organ Transplantation, Nephrology, Dialysis and Transplantation Unit, University of Bari "A. Moro", Bari, Italy

[3]Department of Computer, Control, and Management Engineering (DIAG) Sapienza University, Rome, Italy

[4]Istituto per le Applicazioni del Calcolo "Mauro Picone" - CNR

1 Detailed methodology for network propagation

Typically, the study of the topological structure of protein-protein interactions (PPI) networks, in conjunction with the analysis of several PPI properties, including centrality measures such as degree

centrality, closeness centrality, and betweenness centrality, provide information on the relevance of a specific gene within the PPI. In this work, we deal with seed genes, which are distinguished by a "relevance score" that represents the degree of certainty that a seed gene is relevant for the disease. As a result, classical network measures that provide gene rankings concerning the entire network are not as informative, and classical centrality measures must be extended to provide a rank for seed genes. The purpose of this section is to introduce generalised features and to present the ranking methodology capable of determining the relevance of other genes (nodes) to seed genes and their score.

1.1 Seed genes score

In order to assign the seed genes relevance score, we exploited the DisGeNET gene disease association (GDA) score for those genes belonging to DisGeNET data source that were not manually curated (i.e. examined by an expert gastroenterologist). With regards to the remaining genes (manually curated from PubMed and MEDLINE data sources), we assigned as score the maximum GDA score of the corresponding PBC disease stage (i.e., Early Stages, Late Stages, Unspecified Stages). This choice was made to assign a greater weight to manually curated genes, which, thanks to the specific selection process, can be associated with the disease in a more reliable and robust way. Such genes will have a major impact on the network diffusion process.

1.2 Heat diffusion

The first feature is obtained by using a diffusion process on networks, namely the heat diffusion, that is probably the most used process for disease gene prioritisation and prediction. Starting with a distribution of weights, $\mathbf{s}'(t)$, with positive values only on the seed genes, their evolution is

determined by using the diffusion equation on graph

$$\mathbf{s}'(t) + \mathbf{L}\mathbf{s}(t) = 0\,, \tag{1}$$

where $\mathbf{L}$ is the Graph Laplacian matrix, $\mathbf{L} = \mathbf{D} - \mathbf{A}$, where $\mathbf{D}_{ij} = k_i\delta_{ij}$ is the diagonal matrix with the degree of nodes on the diagonal and $\mathbf{A}$ is the adjacency matrix of the PPI. The weights at time t are given by the formal solution of Eq. (1)

$$\mathbf{s}(t) = \exp\left(-\mathbf{L}t\right)\mathbf{s}(0) \tag{2}$$

where exp is the exponential of the matrix. Several numerical methods provide good approximations of the solution in Equation (2).

Regarding the initial distribution of weights, $\boldsymbol{s}\,(0)$, instead of using a uniform distribution on the seed genes, which is often the typical initial setting, we used the DisGeNET score of each seed gene in order to give more importance to genes with high score, assigning $\mathbf{s}_i(0) = s_i$ for seed genes and 0 otherwise, where s_i is the DisGeNET score i.e.:

$$\mathbf{s}_i(0) = \begin{cases} s_i & \text{if} \quad i \in \Sigma \\ 0 & \text{if} \quad i \notin \Sigma \end{cases} \tag{3}$$

of the seed gene i and Σ is the set of seed genes.

1.3 Balanced diffusion features

The balanced diffusion features are obtained by using another version for the Graph Laplacian matrix, i.e., $\mathbf{L}_b = \mathbf{D}^{-1}\mathbf{I} - \mathbf{A}$. This form of the graph diffusion operator differs from the heat diffusion in the fact that the operator $\mathbf{L}$ diffuses the same amount of score for each link, whereas $\mathbf{L}_b$ diffuses the same amount of score for each node. This implies a different short time behaviour of the diffusion process on the graph. In the first case, the diffusion process proceeds faster from well-connected nodes with high weight, in fact each link carries the same amount of weight and therefore those nodes are drained quickly, whereas in the second case, the same amount of weight diffuses from each node and therefore there is no great difference between well-connected and poorly connected nodes with regard to the outflow of weight.

Similarly to the previous dynamics, the evolution of the weight is determined by using the diffusion equation on graph

$$\mathbf{s}'(t) + \hat{L}_b \mathbf{s}(t) = 0\,. \tag{4}$$

Also in this case the weights at time t are given by the formal solution of Eq. (4)

$$\mathbf{S}(t) = \exp\left(-\hat{L}_B t\right) \mathbf{S}(0) \tag{5}$$

and the initial score is given, as in the heat diffusion, case by Eq. (3).

1.4 NetShort

Another useful feature able to detect an important node with respect to seed nodes is the NetShort measure[1] which is based on the idea that a generic node is topologically important for a disease if a

large number of seed nodes must be traversed to reach it. This concept is implemented establishing that a path between nodes is shorter if it contains more seed nodes compared to other paths. This idea translates in the computation of the average shortest paths reaching the node under consideration. Moreover, to weight the shortest paths favouring links connecting seed nodes and penalising links connecting non-seed nodes, we used the score associated with each seed node giving a weight for each link through the following formula

$$w_{ij} = \mathbf{A}_{ij} \frac{2}{\tilde{s}_i + \tilde{s}_j} \tag{6}$$

where $\tilde{s}_i$ is the normalized score of node i defined as

$$\tilde{s}_i = \begin{cases} \dfrac{s_i}{\max s} & \text{if} \quad i \in \Sigma \\ \alpha \dfrac{\min s}{\max s} & \text{if} \quad i \notin \Sigma \end{cases} \tag{7}$$

where $\min s$ and $\max s$ are the minimum and the maximum of the scores of seed nodes, and α is the penalization parameter given to non-seed nodes. We use $\alpha = 0.5$ so that all non-seed nodes have normalised score $\tilde{s}_i = 0.5\frac{\min s}{\max s}$ while seed nodes have normalised score $\frac{\min s}{\max s} \le \tilde{s}_i \le 1$.

Finally, the NetShort measure of non-seed node i is defined as

$$NS_i = \sum_{j \neq i} \frac{1}{d_{ij}} \tag{8}$$

where d_{ij} is the length of the weighted shortest path from i to j.

1.5 NetRing

The NetRing metric, introduced for the first time in this work, is based on the concept of ring structure [2] generalized to a set of seed nodes. Starting from seed nodes, a partition of the graph in sub-graphs, or rings, is introduced with the following property

$$R(l) \equiv \left\{ j \in V \ \mid \ \min_{i \in \Sigma} l_{ij} = l \right\}. \tag{9}$$

where l_{ij} is the (unweighted) length of the shortest path from i to j. $R(l)$ contains all the non-seed nodes with a minimal distance l from, at least, one seed node. From the definition follows that $R(0) \equiv \Sigma$, $R(l_1) \cap R(l_2) = \emptyset$ if $l_1 \neq l_2$ and $V = \cup_{l=0}^{L} R(l)$, where L is the highest value of the minimal distance from non-seed nodes to seed nodes. Clearly, the concept of ring leads to the introduction of a ranking between nodes, i.e., ring zero includes all seed nodes, ring one includes all the nodes that are directly connected to at least one seed node, and so on as the ring level grows. But, when dealing with seed nodes representing disease genes, it is also evident that not all the nodes in each ring are equivalent, for example there may be a node in the first ring that has only one direct contact with a seed node while another node in the first ring may be directly connected to many seed nodes (it is to be noted that this concept resembles and extends connectivity significance [3]). So, to rank nodes belonging to the same ring, it is important to consider the number of nodes on the lower ring a node is connected to, together with their ranks.

The NetRing metric has been implemented via a two-steps procedure, i) a rank for each seed node starting from its score is initiated: $\hat{r}_i = 1 - \frac{s_i}{\max s}$, ii) the rank of the seed nodes as the convex combination of two terms, the initial rank $\hat{r}_i$, and the average of the initial rank of the neighbors of

the node are defined:

$$r_i(0) = \alpha \hat{r}_i + (1-\alpha)\frac{1}{k_i}\sum_{j|A_{ij}\neq 0} \hat{r}_j\,. \tag{10}$$

In this way the seed nodes that have many other seed nodes as neighbors have the highest ranks.

Then, the rank of non seed-nodes in the ring l, is defined by the following formula:

$$r_i(l) = l + \frac{1}{k_i}\left(\sum_{j\in R_i(l-1)} (r_j(l-1)-(l-1)) + \sum_{j\in O_i} \hat{r}_j\right)\,, \tag{11}$$

namely, it is obtained by summing two terms: 1) the level of the ring, l, 2) the average rank of the neighbors of node i separated in two sets: the first is related to nodes in the previous ring $l-1$ (that we collected in the set $R_i(l-1) = \{j \in R(l-1)|A_{ij} \neq 0\}$) and the second related to the other neighbors of the node i, that can be in the same ring l or in the next ring $l+1$ (we collected them in the set $O_i = \{j \notin R(l-1)|A_{ij} \neq 0\}$). The quantity $(r_j(l-1)-(l-1))$ is the rank of node j inside the ring $l-1$, in order to make a fair ranking with the other neighbors with initial rank $\hat{r}_j$ equal to 1. This measure rewards non-seed nodes that are close to high-scoring nodes in the previous ring and that are linked with a few nodes of the same or higher ring.

1.6 Ranking method

In this section we detail the methodology used to select the most relevant non-seed genes for the disease using the features that have been introduced in the previous paragraphs. The methodology is based on a positive-unlabelled algorithm. Starting from the definition of positive samples, P, namely the seed genes, and defining reliable negative samples, RN, namely genes that are likely not related to the disease, through an algorithm of label propagation, we identify likely-positive samples, LP, namely non-seed genes that are likely to be linked to the disease.

Let V be a set whose generic element v_i, for $i = 1, ..., n$, is characterised by the couple $(\mathbf{x}_i, y_i)$ where $x_i \in [0,1]^d$ represents the features vector (obtained using the features described in the previous sections) and $Y_i \in -1, 0, 1$ is the initial label, where 1 is associated to seed genes (set P), -1 to reliable negative genes (set RN), and 0 to uncharacterised genes. The label propagation process can be defined by the following Steps:

Step 1: compute the matrix $\boldsymbol{W}$, whose elements w_{ij}, with values between 0 and 1, represents the similarity score between elements i and j, defined as follows

$$w_{ij} = \begin{cases} 1 - \frac{e_{ij} - m}{M - m} & \text{if } i \neq j \\ 1 & \text{otherwise} \end{cases} \tag{12}$$

where e_{ij} is the euclidean distance between the features of elements i and j, $e_{ij} = \sum_k \left(x_i^k - x_j^k\right)^2$, $m = \min_{ij} \{e_{ij}\}$ and $M = \max_{ij} \{e_{ij}\}$. The present definition of $\boldsymbol{W}$ preserve the symmetry of the similarity score between the elements i and j;

Step 2: Compute the reduced matrix $\boldsymbol{W}_r$ as follows

$$w_{r,ij} = \begin{cases} w_{ij} & \text{if } w_{ij} > s \\ 0 & \text{otherwise} \end{cases}. \tag{13}$$

where all the elements of $\boldsymbol{W}$ below the threshold score s are set to zero. The threshold s is computed as a given quantile of the distribution of the elements in the matrix $\boldsymbol{W}$ (for example the quantile 0.05 can be used as threshold), in order to exclude from the label propagation process links between elements that are poorly related. The matrix $\boldsymbol{W}_r$ is then normalised as follows

$$\boldsymbol{W}_n = \boldsymbol{D}^{-1} \boldsymbol{W}_r \tag{14}$$

where $\boldsymbol{D}$ is the diagonal matrix with elements $D_{ii} = \sum_j w_{r,ij}$.

Step 3: compute the average feature of seed genes,$\mathbf{x}_s$, as $x_s^k = \sum_{i|y_i=1} x^k|P|$, where —P— is the number of seed genes. Then, reliable negative genes, RN, are selected as those genes with features that are the furthest from $\mathbf{x}_s$. In order to create a balanced sample, we select exactly the —P— most distant genes from the average feature $\mathbf{x}_s$. We initialise the label propagation process with the vector G_0, that contains the initial labels, set as 1 for elements in P, -1 for elements in RN, and 0 for the remaining elements.

Step 4: Label propagation. Starting from vector G_0, a Markov process with restart is introduced in order to obtain an iterative stationary distribution of propagating labels:

$$G_r = (1 - \alpha)\, \boldsymbol{W}_n^{\boldsymbol{t}} G_{r-1} + \alpha G_0 \tag{15}$$

where the parameter α is usually set to 0.8 [4, 5]. This process guarantees the conservation of the sum of the elements of G_r for each r, and that the positive and negative values diffuse to their neighbors (with probability $(1 - \alpha)$) and restart from the initial distribution (with probability α) till a stationary distribution is considered reached when $|G_r - G_{r-1}| < 10^{-6}$. The asymptotic vector is called G_∞.

Step 5: The likely positive genes are chosen as those genes having the highest weight once the seed genes are excluded.

References

[1] Scott White and Padhraic Smyth. Algorithms for estimating relative importance in networks. In *Proceedings of the ninth ACM SIGKDD international conference on Knowledge discovery and data mining*, pages 266–275, 2003.

[2] Andrea Baronchelli and Vittorio Loreto. Ring structures and mean first passage time in networks. *Physical Review E*, 73(2):026103, 2006.

[3] S. D. Ghiassian, J. Menche, and A. L. Barabási. A DIseAse MOdule Detection (DIAMOnD) algorithm derived from a systematic analysis of connectivity patterns of disease proteins in the human interactome. *PLoS Comput Biol*, 11(4):e1004120, Apr 2015.

[4] Peng Yang, Xiao Li Li, Jian Ping Mei, Chee Keong Kwoh, and See Kiong Ng. Positive-unlabeled learning for disease gene identification. *Bioinformatics*, 28:2640–2647, 2012.

[5] Yongjin Li and Jagdish C Patra. Genome-wide inferring gene–phenotype relationship by walking on the heterogeneous network. *Bioinformatics*, 26(9):1219–1224, 2010.
