# Supplementary figures and images for: Network Proximity-Based Drug Repurposing Strategy for Early and Late Stages of Primary Biliary Cholangitis
Source: Biomedicines. 2022 Jul 13;10(7):1694. doi: 10.3390/biomedicines10071694 (PMC9312896; doi:10.3390/biomedicines10071694)

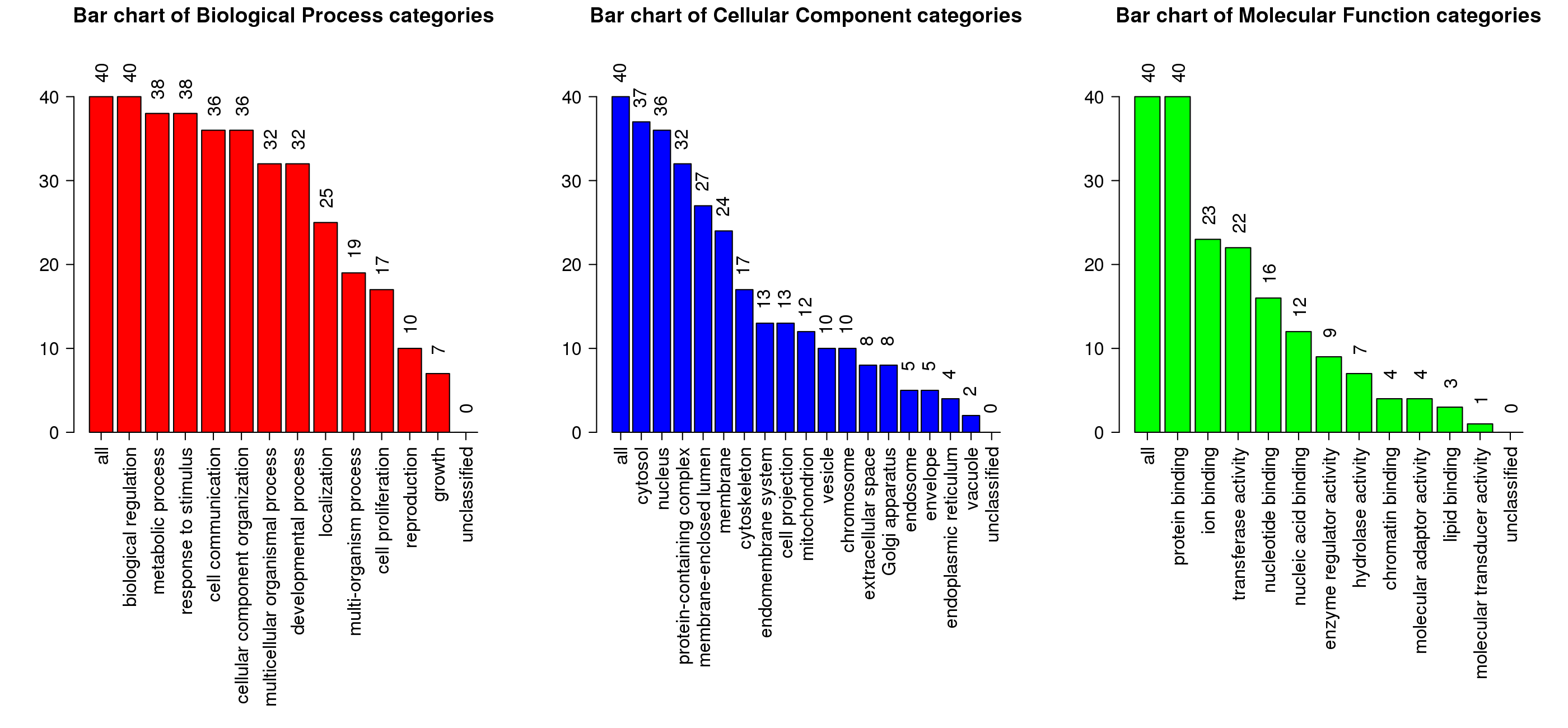

Supplement: Supplementary file 1 [file biomedicines-10-01694-s001.zip › Supplementary files S1 to S7/Supplementary File S2_l-threonine_/goslim_summary_wg_result1653302526.png]

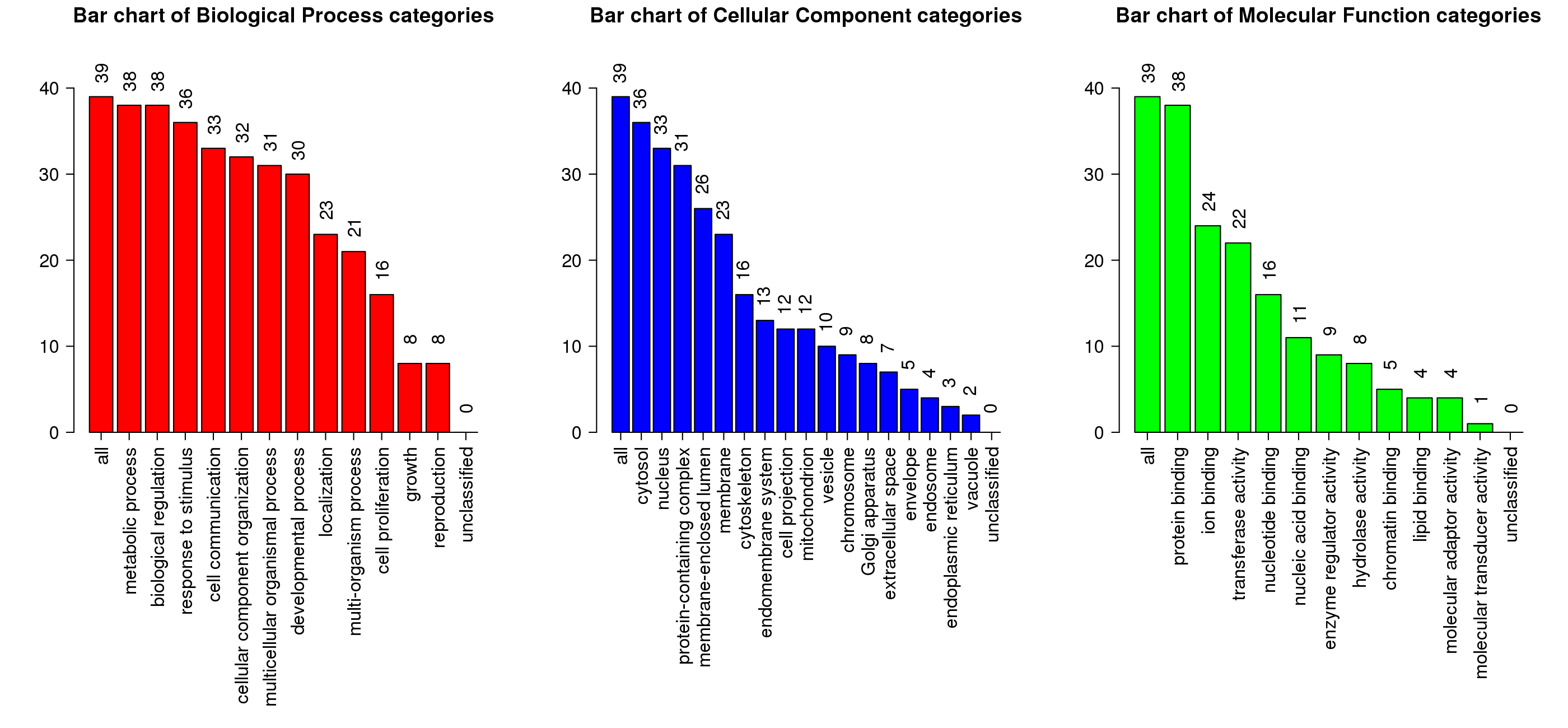

Supplement: Supplementary file 1 [file biomedicines-10-01694-s001.zip › Supplementary files S1 to S7/Supplementary File S3_l-serine/goslim_summary_wg_result1653302713.png]

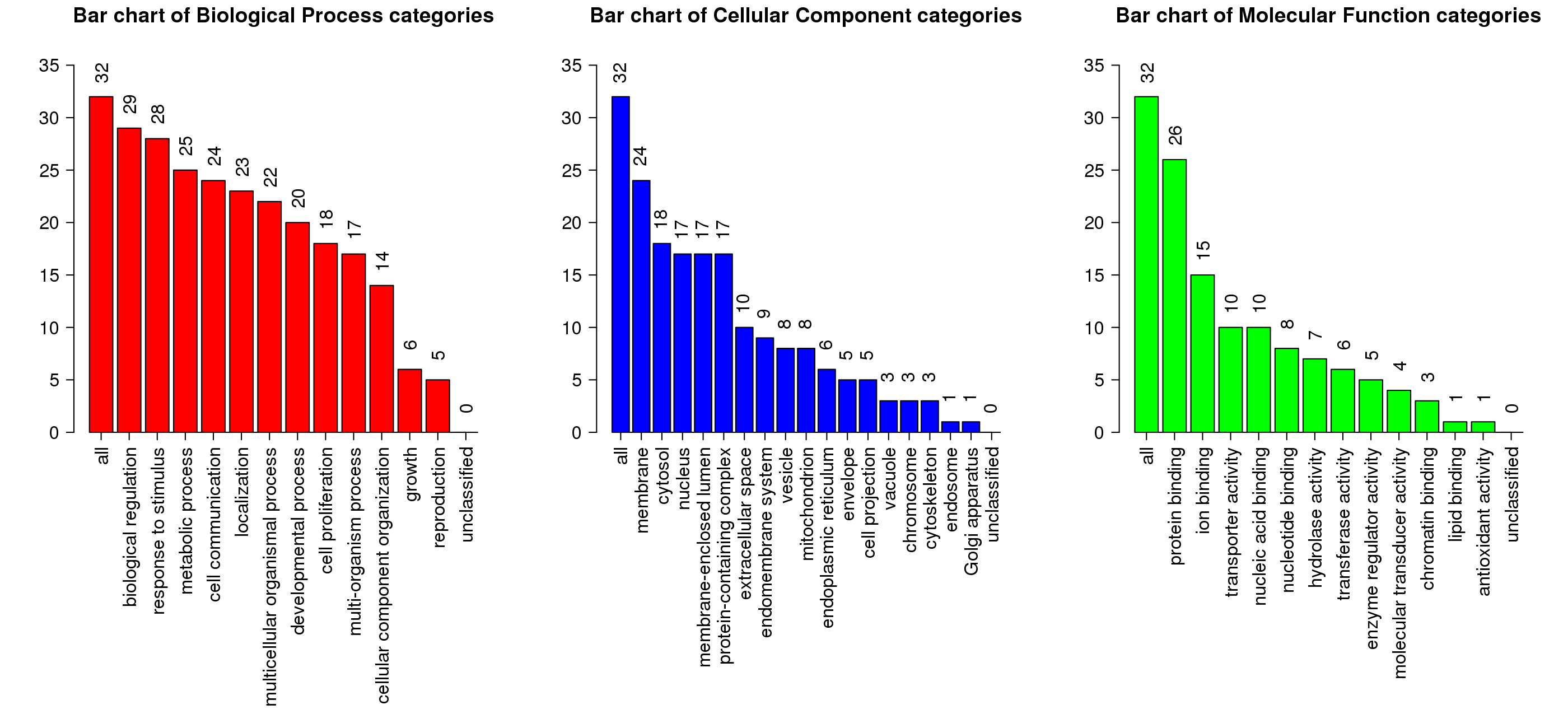

Supplement: Supplementary file 1 [file biomedicines-10-01694-s001.zip › Supplementary files S1 to S7/Supplementary File S5_PA452621/goslim_summary_wg_result1653310428.png]

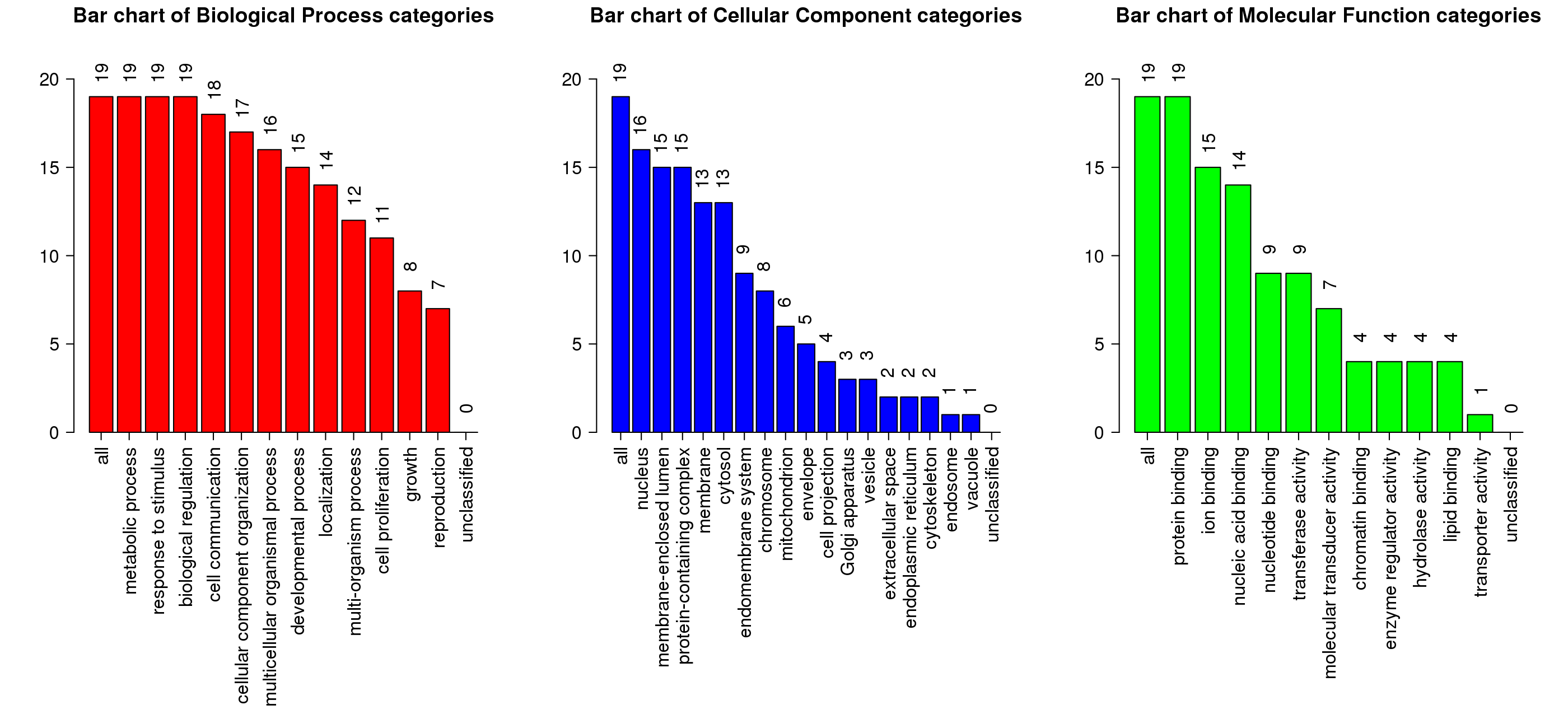

Supplement: Supplementary file 1 [file biomedicines-10-01694-s001.zip › Supplementary files S1 to S7/Supplementary File S6_PA164712966/goslim_summary_wg_result1653310882.png]

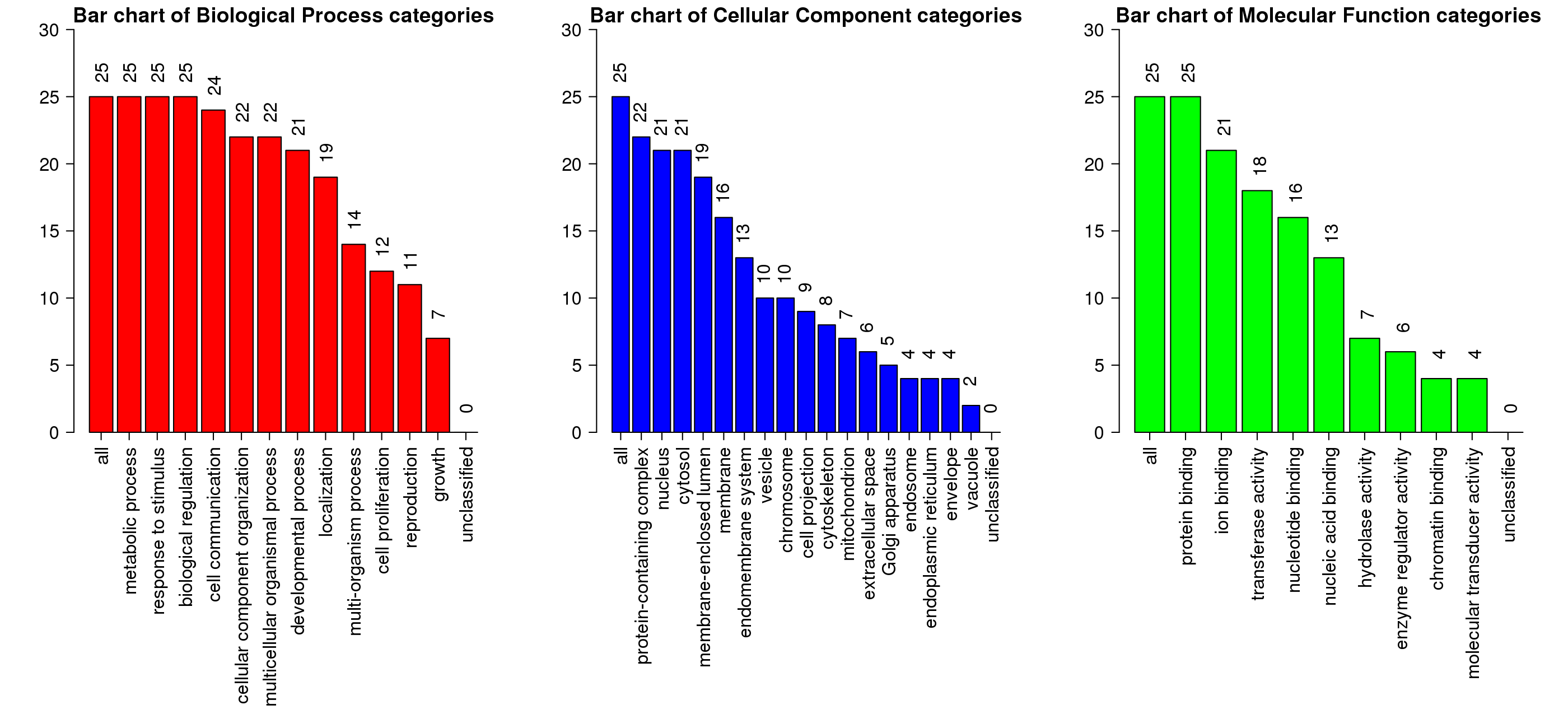

Supplement: Supplementary file 1 [file biomedicines-10-01694-s001.zip › Supplementary files S1 to S7/Supplementary File S7_PA164712732/goslim_summary_wg_result1653313265.png]

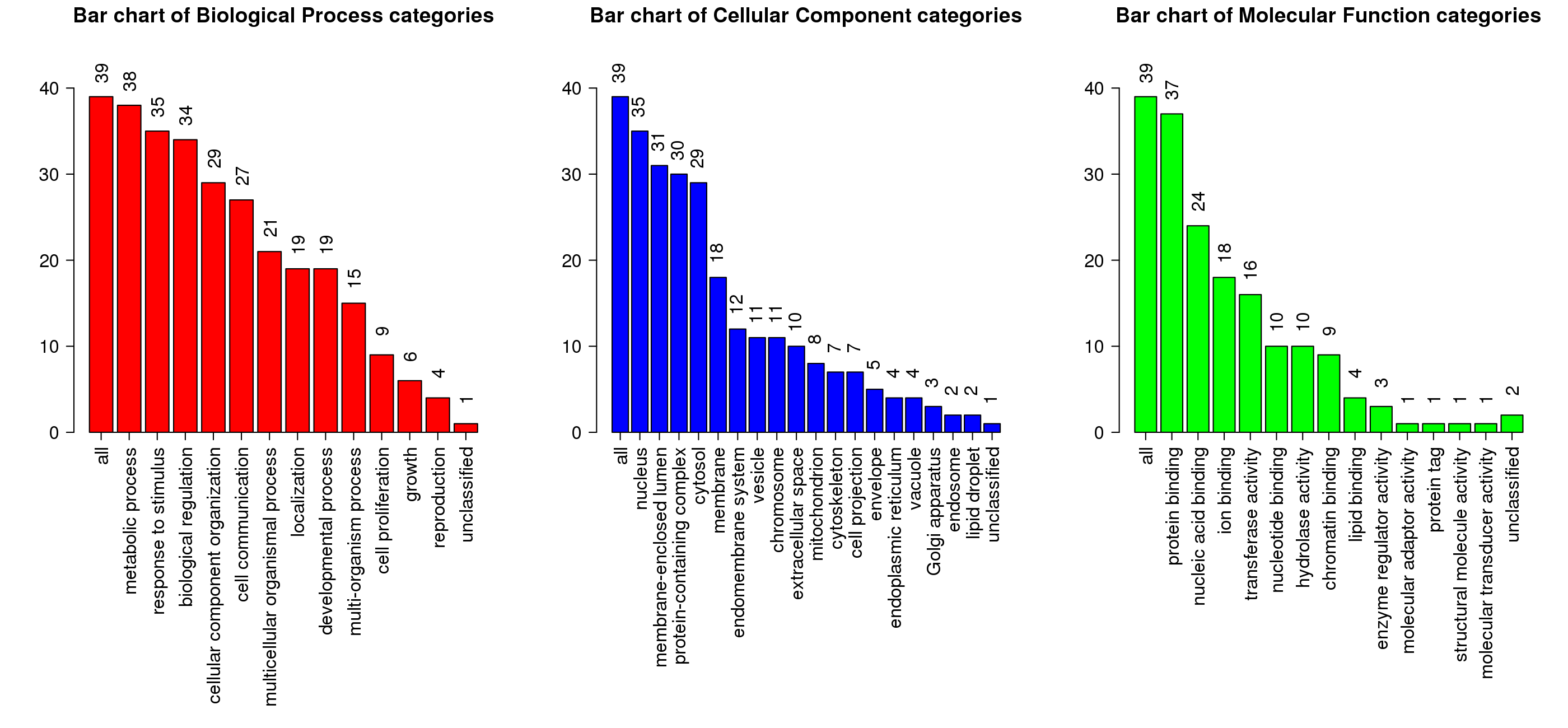

Supplement: Supplementary file 1 [file biomedicines-10-01694-s001.zip › Supplementary files S1 to S7/Supplementary File_S4_l-lysine/goslim_summary_wg_result1653302621.png]
